# Mechanisms of Vitamin C Regulating Immune and Inflammation Associated with Neonatal Hypoxic-Ischemic Encephalopathy Based on Network Pharmacology and Molecular Simulation Technology

**DOI:** 10.1155/2022/4904325

**Published:** 2022-02-14

**Authors:** Shangbin Li, Shuangshuang Li, Qian Zhao, Jiayu Huang, Jinfeng Meng, Weichen Yan, Jie Wang, Changjun Ren, Ling Hao

**Affiliations:** ^1^The First Hospital of Hebei Medical University, Shijiazhuang, Hebei, China; ^2^Chengdu University of Traditional Chinese Medicine, Chengdu, China

## Abstract

**Background:**

There are still controversies about the curative effect of vitamin C in treating HIE, and its mechanism of action is not entirely clear. This study is designed to explore the potential molecular mechanism of vitamin C in treating neonatal hypoxic ischemic encephalopathy (HIE).

**Methods:**

The effect targets of vitamin C and the pathogenic targets of neonatal HIE were obtained via retrieval of public databases to screen out the molecular targets of vitamin C acting on neonatal HIE. Gene Ontology (GO) functional annotations and Kyoto Encyclopedia of Genes and Genomes (KEGG) pathway analysis were performed on the main targets. Vitamin C and the optimum target structural components are subjected to molecular docking and molecular dynamics simulation analysis via computer software so as to verify their binding activity and stability.

**Result:**

Based on 16 overlapping targets of vitamin C and HIE, seven main targets were identified in this study. According to GO and KEGG analysis, molecular functions (top 25 items) and signal pathways (21 items) related to inflammatory reaction, immune response, and cell transcriptional control may be potential pathways for vitamin C to treat neonatal HIE. Molecular docking and molecular dynamics simulation were adopted to definitively determine the 4 optimum core target spots.

**Conclusion:**

The efficacy of vitamin C on HIE is involved in the immunoregulation and inflammation-related functional processes and signal pathways. These molecular mechanisms, including core targets, will contribute to the clinical practice of neonatal HIE in the future.

## 1. Introduction

Hypoxic ischemic encephalopathy (HIE) is a brain damage caused by asphyxia at birth, as well as one of the most common causes of neonatal death and long-term disability, occurring in 2 to 3 per 1000 newborns [1]. Despite the timely adoption of hypothermia therapy, there are still unacceptable complications in some newborns with severe perinatal asphyxia, which bring a significant burden to global public health problems and society [2, 3]. By now, the pathological and biochemical changes of neonatal hypoxic-ischemic brain damage are a cascade process, with nerve cell apoptosis or necrosis being the final result of brain damage [4]. Many studies have shown that the central nervous system will produce large amounts of oxygen radicals and inflammatory mediators that promote nerve cell necrosis and apoptosis in the hours following an hypoxic ischemic brain damage and during brain reperfusion and reoxygenation [5, 6]. In addition, reactive oxygen radicals have been proven to be the signal transduction mediators of transcriptional factors that lead to apoptosis after cerebral ischemia [7]. Some in vitro studies have found that intervention in the early inflammatory phase of hypoxic ischemic brain damage can protect neurons to reduce the degree of brain damage [8, 9]. Therefore, some scholars suggest that attention should be paid to the role of antioxidant and anti-inflammatory interventions in the treatment of neonatal hypoxic ischemic brain damage on the basis of mild hypothermia therapy [10].

Many anti-inflammatory and antioxidant potential drugs have been studied in preterm birth and term hypoxic ischemic brain damage, such as erythropoietin, melatonin, and histone deacetylase inhibitors, which have shown potential neuroprotective effects in animal models [9, 11, 12]. However, only a few studies have progressed to clinical trials or failed to show the desired results in clinical trials. As one of the best drug candidates that affect the pharmacology of the brain damage stage, vitamin C is a micronutrient that can protect neurons from oxidative stress, reduce inflammation, regulate neurotransmission, influence neuronal development, and control epigenetic inheritance [13–15]. In addition, vitamin C acts as a cofactor in several important enzyme reactions [16]. Recently, a growing number of scholars are committed to the study of vitamin C as an adjuvant therapy for a variety of diseases, especially in adults with central nervous system diseases such as ischemic stroke, Alzheimer's disease, and Parkinson's disease [17–19]. As for neonatal hypoxic ischemic brain damage, animal studies have shown that low-dose systemic injection of vitamin C can increase the concentration of vitamin C in the brain tissue, thereby taking a significant neuroprotective effect on HIE [20]. Studies have indicated that vitamin C adjuvant therapy for HIE can improve the relevant antioxidant and anti-inflammatory indexes in the blood of pediatric patients and obtain better curative effects [21]. However, few studies have found no significant clinical efficacy, which cannot be ruled out as a result of the dose of vitamin C, route of administration, or other mechanisms in clinical trials for HIE [22, 23]. At present, there is still controversy regarding the clinical efficacy of vitamin C in treating HIE. Furthermore, the details of the biological targets and signal pathways in which it functions are still unclear, and in-depth institutional preclinical studies are limited.

In recent years, as a part of bioinformatics technology, network pharmacology has unprecedented advantages with the rapid development of bioinformatics technology, especially molecular docking, molecular dynamics, and other computer simulation technologies [24]. On the one hand, it can explore the relationship between drugs and diseases as a whole, and on the other hand, it can systematically reveal the molecular mechanism of drug-disease interactions. If those technologies are applied to the field of pediatrics, it is expected to deepen the understanding of the mechanism of disease and the mechanism of drug action [25].

Therefore, this study aimed to use this emerging approach of bioinformation technology to decipher the predicted targets and signaling pathways of vitamin C's role in HIE so as to provide a scientific basis for future clinical practice and experimental research.

## 2. Methods

### 2.1. Screening of Vitamin C-Disease Targets

Traditional Chinese Medicine System Pharmacology (TCMSP, http://lsp.nwu.edu.cn/index, accessed July 18, 2021) [26], Drugbank (https://www.drugbank.com/, accessed July 18, 2021) [27], Bind DB (http://bindingdb.org/bind/index, accessed July 18, 2021) [28], and Swiss Target Prediction (https://www.swisstargetprediction.ch/, accessed July 18, 2021) [29] databases were applied to retrieve researched and reported vitamin C pharmacological targets with “vitamin C” and “ascorbic acid” as keywords. In the same way, DisGeNET (http://www.disgenet.org, accessed July 20, 2021) [30], DrugBank (https://www.drugbank.com/, accessed July 20, 2021), and GeneCards (https://www.genecards.org/, accessed July 20, 2021) [31] databases were used to screen and get the pathogenic genes of neonatal HIE, with “Neonatal HIE” and “Neonatal Hypoxic Ischemic Brain Damage” as keywords [32]. Then, the UniProt database (https://www.UniProt.org/, accessed July 23, 2021) [33] was applied to standardize gene names, and the Venn diagram was used to screen out common targets for vitamin C and neonatal HIE.

### 2.2. Screening of Optimum Core Targets of Vitamin C on HIE and Construction of Interrelated Network

The STRING database (https://string-db.org/, accessed July 23, 2021) was used to obtain the protein-protein interaction (PPI) between target-to-target functions [34]. The common targets between vitamin C and neonatal HIE were input to construct PPI network models using biological species set to “*Homo sapiens*”; nodes with degree of confidence >0.4 and unconnected nodes were hidden; the remaining parameters are set to default values. Related data obtained were downloaded in TSV file format, and the Cytoscape v3.7.1 software (http://www.cytoscape.org/, accessed April 15, 2021) was used to set up the PPI network [35]. Stable structures in the network were filtered by means of the MCODE plug-in [36]. The MCODE plug-in was used to set up functional modules, and the module filtering criteria are degree cutoff = 5, node score = 0.2, K-core = 2, and max depth = 100. The optimum target was determined based on the best score of the module [37, 38].

### 2.3. Analysis of Molecular Functional Enrichment and Signaling Pathways

The enrichment analysis was performed for the previously determined optimum target by the R package of “ClusterProfiler” on the biological process (BP), cellular component (CC), molecular function (MF) of gene ontology (GO), and Kyoto Encyclopedia of Genes and Genomes (KEGG) pathways [39, 40]. All data were visualized with histograms or bubble diagram [41]. The enrichment data were obtained from “org.Hs.eg.Db”, and *P* < 0.05 was used as the cutoff value for significant enrichment after adjustment.

### 2.4. Molecular Docking Verification

Molecular docking technology was used to study vitamin C and its related targets for the treatment of HIE, which can explain the mechanism and binding activity of vitamin C and target proteins to a certain extent [42]. The structure of the compound in SDF format was downloaded from the PubChem database (https://pubchem.ncbi.nlm.nih.gov/, accessed July 26, 2021), and the Chem 3D software was used to convert the SDF format into a mol2 format file. The structure of the related protein in PDB format was downloaded from the RCSB database (https://www.rcsb.org/, accessed July 26, 2021); the PyMOL software (https://pymol.org/2/, accessed July 27, 2021) was applied to remove solvent molecules and ligands, and AutoDock Tools v1.5.6 was used for software hydrogenation, calculation of charge, allocation of atom types, and so on., and saved in pdbqt format. Finally, the AutoDock Vina v1.1.2 software was run to perform molecular docking, and conformations with binding free energy less than −5 kcal/mol were selected for docking and visual analysis via the Discovery Studio 2020 software (https://www.3ds.com/, accessed December 25, 2020).

### 2.5. Molecular Dynamics Simulation

Gromacs is a powerful molecular dynamics simulation software, which has great advantages in simulating the Newtonian motion of a large number of molecular systems [24]. The molecular dynamics (MD) simulation uses the Gromacs 2018.4 program [43] to perform the molecular dynamics simulation on the protein active ingredients obtained by molecular docking under constant temperature and pressure and periodic boundary conditions. Amber99SB all-atom force field and TIP3P water model were applied [44]. In the MD simulation process, all bonds involving hydrogen atoms were constrained by the LINCS algorithm [45], and the integration step was 2 fs. Electrostatic interaction was calculated via the PME (Particle-mesh Ewald) method [46]. The cutoff value for nonbonding interactions was set to 10 Å, which was updated every 10 steps. The V-rescale temperature coupling method [47] was conducted to control the simulated temperature to 300 K, and the Parrinello–Rahman method [48] was used to control the pressure to 1 bar. Firstly, the steepest descent method was applied to minimize the energy of the system in the vacuum environment to eliminate the overclose contact between atoms. The maximum number of iterations in the energy optimization process was set as 5000, and the convergence value of energy minimization was 100 kJ·mol^−1^·nm^−1^. Then, the NVT and NPT equilibria were simulated at 300 K for 1 ns, respectively. Finally, a 100 ns MD simulation was performed on the system, and the conformation was saved every 10 ps. The simulation results were visualized using the Gromacs embedded program and VMD software (https://www.ks.uiuc.edu/Research/vmd/, accessed December 28, 2020).

### 2.6. Visualization of Vitamin C Active Target-Disease-Related Network Relationships

In order to further clarify their mechanism of action, the Cytoscape v3.7.1 software was applied to construct an intuitive relationship network via setting the optimal targets as the source node and the signaling pathway as the target node; next, the results of the finally determined stable binding targets with vitamin C and KEGG signal pathways were visualized [49]. In addition, the final optimal targets were imported into the National Center for Biotechnology Information platform (https://www.ncbi.nlm.nih.gov/gds, accessed August 2, 2021) and converted into gene-IDs; then, the corresponding gene-ID was imported into the KEGG Mapper platform (https://www.genome.jp/kegg/mapper/, accessed August 2, 2021) that identified the relevant pathways [50].

## 3. Results

### 3.1. Detailed Data of Targets

**Figure 1:**
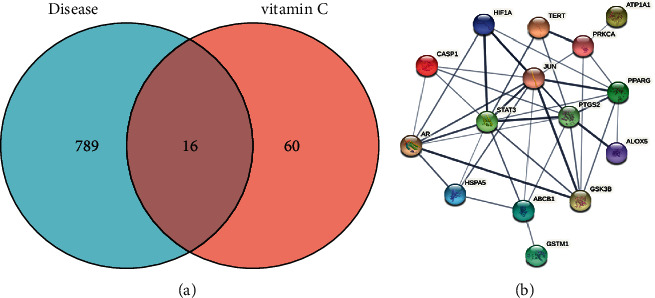
(a) Venn figure and (b) protein-protein interaction network.

The HIE related genes of newborns were screened through the DisGeNET database; screening criteria: gene-disease association score >0.1; the HIE of newborns was screened through the GeneCards database; screening criteria: gene score >1.805; genes related to neonatal HIE were determined with the above criteria. Vitamin C drug targets were screened and identified via TCMSP, Bind DB, DrugBank, and Swiss Target Prediction databases, and 76 vitamin C targets were identified via the UniProt database. Then, 16 overlapping targets (Table 1, Figure 1(a)) were obtained by using the Venn diagram intersection. Credibility of 0.400 was set as the minimum interaction score. PPI data related to the function of these 16 targets were collected via the STRING database, and a PPI network related to the pharmacological targets and functions of vitamin C on neonatal HIE was then constructed (Figure 1(b)).

### 3.2. PPI Network Topology Parameters and the Optimum Core Targets

**Figure 2:**
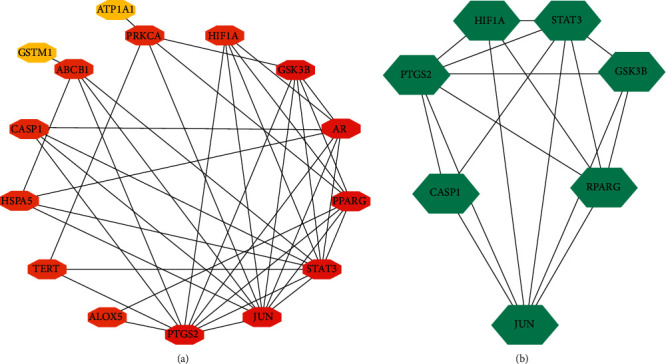
(a) The arrangement network of the PPI in Cytoscape. (b) The screened module network.

The PPI data obtained from the STRING database were imported into the Cytoscape software, and topological parameters of the interaction network between vitamin C and HIE with common targets and function-related proteins were calculated (Figure 2(a): 15 nodes and 40 edges). The MCODE plug-in was used to obtain a key module by *k*-value screening (Figure 2(b): module score = 5.667), and the genes in the module also had a high connectivity. These genes were believed to play a key role in the development of HIE and deserve further study. Therefore, the genes in the module were defined as key core targets, including signal transducer and activator of transcription 3 (STAT3), prostaglandin G/H synthase 2 (PTGS2), transcription factor AP-1 (JUN), and peroxisome proliferator-activated receptor *γ* (PPARG), glycogen synthase kinase-3*β* (GSK3B), cysteine protease-1 (CASP1), and hypoxia-inducible factor 1-*α* (HIF1A).

### 3.3. GO Function Analysis and KEGG Pathway Enrichment Analysis

**Figure 3:**
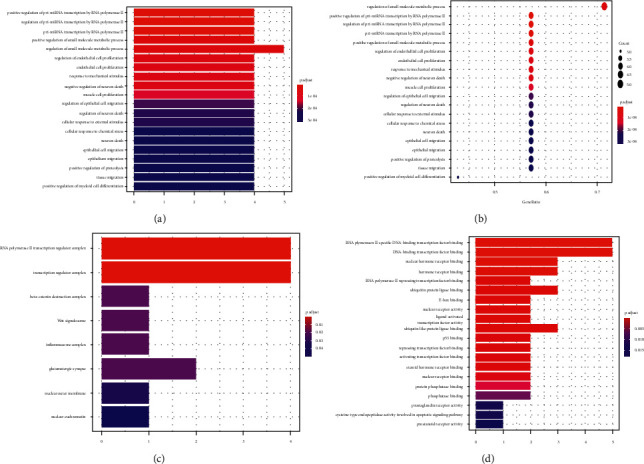
GO enrichment analysis of the core target of vitamin C in the treatment of HIE (the top 25 items). (a, b) biological processes, (c) cell components, and (d) molecular functions.

**Figure 4:**
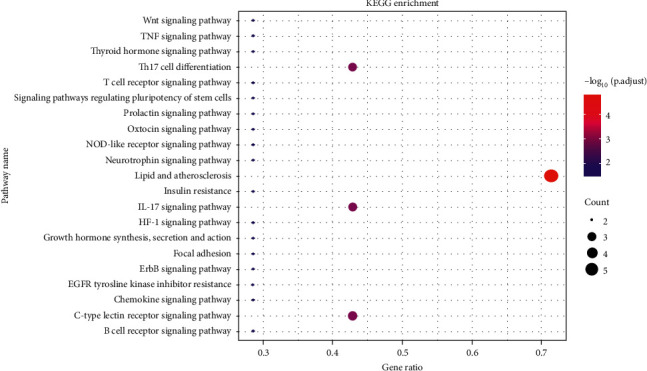
KEGG enrichment analysis of vitamin C in the treatment of HIE (21 items).

GO enrichment and KEGG pathway analysis were performed on 7 key targets via R language software packages. According to the results of the study, the biological processes related to these targets (Figures 3(a) and 3(b)) mainly included the regulatory influence of RNA polymerase II on pri-miRNA transcription, the regulation of small molecule metabolic processes, the response to mechanical stimuli, and the effects of epithelial cell migration, regulation of small molecule metabolism, response to mechanical stimulation, regulation of epithelial cell migration, regulation of neuronal death, cell response to external stimuli, regulation of angiogenesis, positive regulation of endothelial cell proliferation, and regulation of inflammatory response. Cellular components related to these targets (Figure 3(c)) included RNA polymerase II transcription regulatory complex, transcriptional regulatory complex, *β*-catenin destruction complex, Wnt signal body, inflammasome complex, glutamatergic synapse, nuclear outer membrane, and nuclear euchromatin. In terms of molecular function (Figure 3(d)), vitamin C used to treat HIE mainly involves the regulation of nuclear hormone receptor binding, hormone receptor binding, RNA polymerase II inhibits transcription factor binding, nuclear receptor binding, ligand-activated transcription factor activity, ubiquitin-like protein ligase binding, p53 binding, and regulatory transcription factor binding. KEGG enrichment analysis yielded a total of 41 significantly enriched pathways, and the results were screened to obtain 21 closely related pathways (Figure 4), mainly including immune inflammation pathways such as the IL-17 signaling pathway, C-type lectin receptor signaling pathway, Th17 cell differentiation, B cell receptor signaling pathway, tumor necrosis factor signaling pathway, and T cell receptor signaling pathway; hormone metabolism pathways such as prolactin signal pathway, insulin resistance, growth hormone synthesis, and secretion, and hypoxic stress pathways such as HIF-1 signal pathway. In general, GO function enrichment analysis and KEGG signal pathway enrichment results mainly involved inflammation, immune response, and cell transcription regulation. Other BP, CC, MF, and KEGG pathways were listed in the supplementary file.

### 3.4. Molecular Docking of Vitamin C-Targets

**Figure 5:**
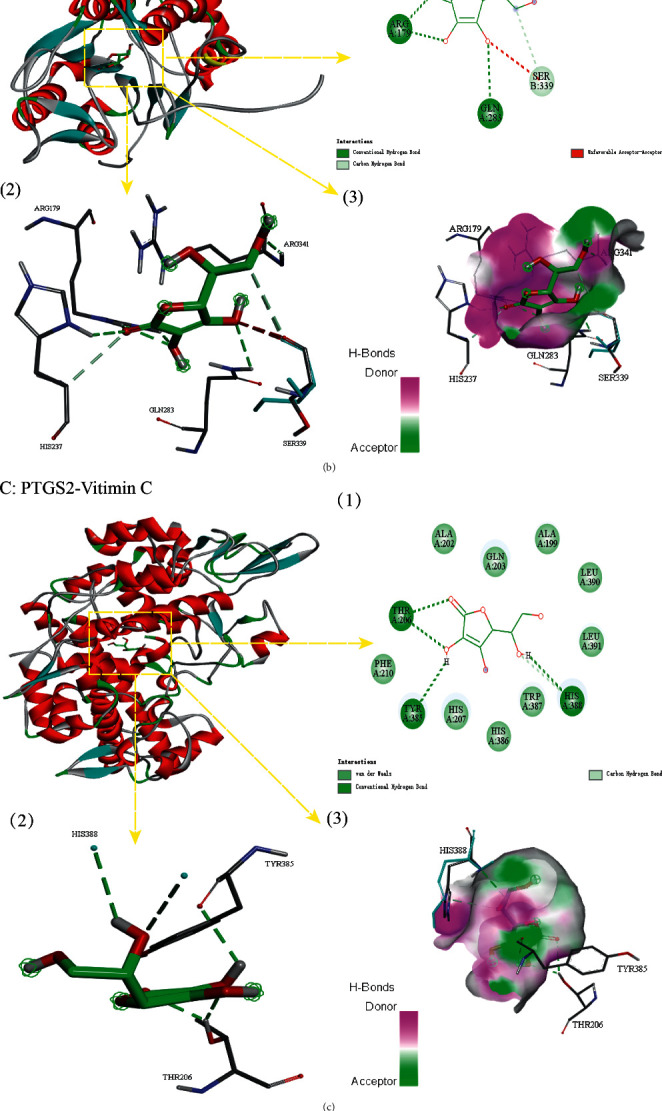
The docking model of vitamin C and 4 core target molecules. (a) HIF1A-vitamin C. (b) CASP1-vitamin C. (c) PTGS2-vitamin C. (d) PPARG-vitamin C.

The AutoDock Vina tool was used to conduct semiflexible molecular docking between the module gene and vitamin C in the PPI network to verify the binding activity of vitamin C and the target. Generally speaking, the combination of small and large molecules is mainly evaluated by binding energy. Generally, a binding energy of less than 0 kcal·mol^−1^ indicates that there is a binding activity between molecules, and a binding energy of less than −5.0 kcal/mol indicates that there is a strong binding activity between molecules [51]. The final results showed that vitamin C did indeed bind well to HIF1A, CASP1, PTGS2, and PPARG (Table 2, Figure 5). The lowest binding free energy is with HIF1A-vitamin C (−6.8 kcal/mol), and the main forces are hydrogen bonds and van der Waals forces. This was followed by the vitamin C-CASP1 (−6.5 kcal/mol), which formed hydrogen bond interactions with amino acids HIS 237, ARG 179, GLN 283, and ARG 341 and interacted carbon-hydrogen bonds with amino acid SER 339 to maintain a stable conformation together. The third is vitamin C-PTGS1 (−6.3 kcal/mol), both of which maintain a stable conformation together by forming hydrogen bonds or van der Waals interactions with amino acids such as THR 306, TYR 385, HIS 388, and PHE 210; the last one is vitamin C-PPARG, which formed hydrogen bonds or van der Waals interactions with amino acids such as TYR 22, ILE 296, and LEU 228 and maintained their stable conformation together by forming *π*-cations or hydrocarbons with amino acids MET 329, PRO 227, and SER 225.

### 3.5. Molecular Dynamics Simulation

**Figure 6:**
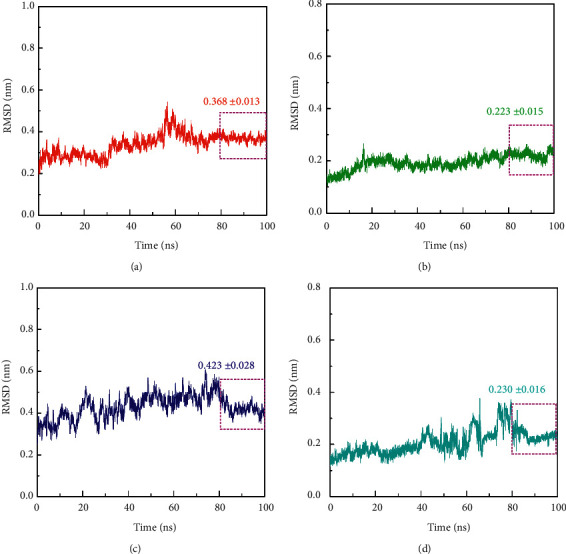
RMSD diagram during molecular dynamics simulation. (a) RMSD of HIF1A-vitamin C. (b) RMSD of PTGS2-vitamin C. (c) RMSD of CASP1-vitamin C. (d) RMSD of PPARG-vitamin C.

The core targets were selected for molecular docking, and the molecular dynamics simulation of 100 ns was performed with vitamin C. The root mean square deviation represents the sum of all atomic deviations between the conformation at a certain time and the target conformation, which was an important basis for measuring the stability of the system and can be used to quantify the stability of the tertiary structure of the complex formed by small protein molecules. Therefore, this paper selected the core target determined by molecular docking and vitamin C to perform a 100 ns molecular dynamics simulation and checked the system stability by calculating the root mean square deviation (RMSD) within 100 ns of the simulated trajectory. At the beginning of the simulation, there was a preliminary upward trend, which was mainly due to the interaction between the protein and the surrounding water solvent at the beginning of the simulation, causing certain fluctuations in the protein structure. As shown in Figure 6, the RMSD change trend of vitamin C and the four protein complexes was the same. On the whole, the RMSD values of the four systems were stable in the last 20 ns. The RMSD value of the first system was 0.368 ± 0.013 nm,the RMSD value of the second system was 0.223 ± 0.015 nm, the RMSD value of the third system was 0.423 ± 0.028 nm, and the RMSD value of the fourth system was 0.230 ± 0.016 nm. The overall RMSD fluctuations of the four complexes were all less than 0.3 nm, indicating that the four complex systems were relatively stable during the simulation process.

**Figure 7:**
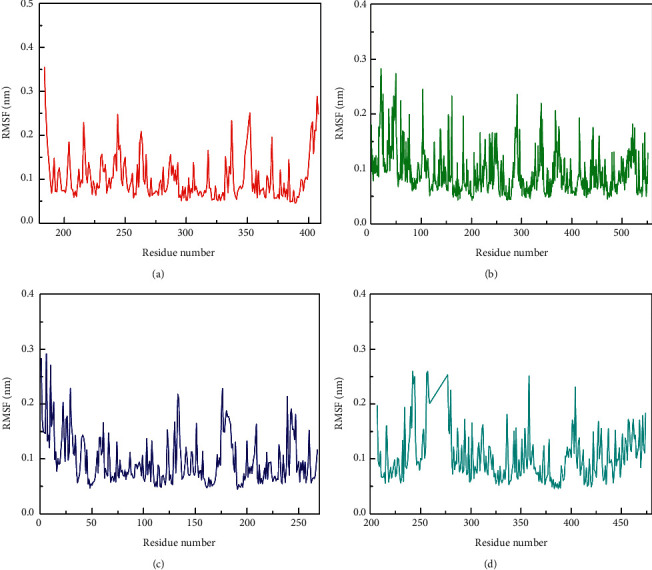
RMSF diagram during molecular dynamics simulation. (a) RMSF of HIF1A-vitamin C. (b) RMSF of PTGS2-vitamin C. (c) RMSF of CASP1-vitamin C. (d) RMSF of PPARG-vitamin C.

Root mean square fluctuations can indicate the flexibility of amino acid residues in a protein. As shown in Figure 7, the analysis results of the protein amino acid RMSF in the four complex systems showed that the overall amino acid flexibility was less than 0.3 nm, and the binding of vitamin C did not significantly affect the protein amino acid flexibility, indicating that vitamin C could stably bind in all the four active pockets of the protein without changing the protein structure.

### 3.6. The Construction of Vitamin C-Target-KEGG-HIE Network Graph

**Figure 8:**
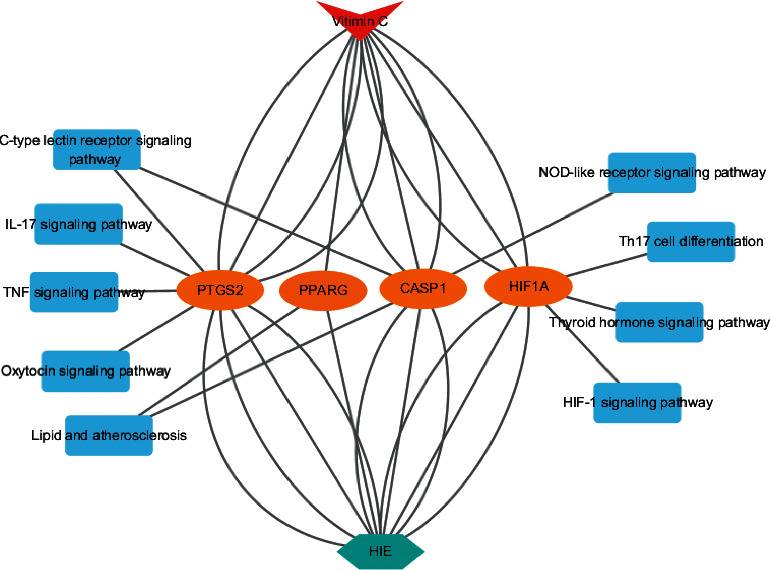
Visualization of the vitamin C-target-KEGG-HIE network diagram.

**Figure 9:**
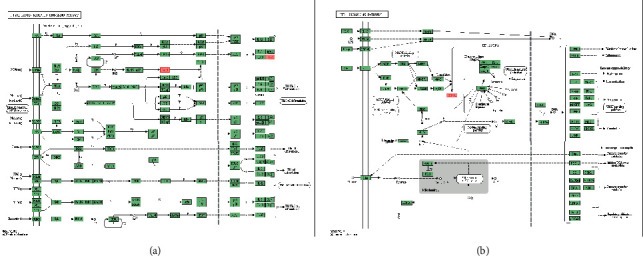
Signaling pathway diagram. (a) Type C lectin receptor signaling pathway. (b) HIF-1 signaling pathway.

The Cytoscape software was used to import the optimal target and the signal pathway that intersected with the target, and the interaction diagram of vitamin C's core target-related pathways in HIE was generated (Figure 8), which further clarified the potential molecular mechanism of vitamin C in HIE from the perspective of the system. The same target could intervene in different signal pathways as indicated via the interactive network. In addition, the KEGG Mapper tool was used to identify some related signaling pathways (Figure 9). The optimal target from interlaced networks identified through KEGG Mapper assays were highlighted in red, and it was found that some upstream pathways could regulate downstream pathways. Other relevant pathways were listed in the supplementary table.

## 4. Discussion

At present, there is no unified standard and specialized treatment for HIE. In clinical practice, the challenge is that a gap remains between preclinical medical research and clinical application in the treatment of neonatal HIE [52]. Vitamin C, as an antioxidant, interacts with important transcription factors to regulate gene expression [53]. At present, however, there is no report on the target and signal mechanism of vitamin C in treating HIE. Therefore, it is a potential research approach for systematic network pharmacology to be used to identify and optimize the screening of biological targets, functional processes, and signaling pathways that vitamin C plays a role in treating HIE, which can be performed before preclinical medical research and clinical application.

Based on bioinformatics analysis, this study efficiently obtained all potential biological targets of the effect of vitamin C on HIE and predicted 7 key pharmacological targets via topology analysis. Molecular docking technology confirmed that vitamin C has a good binding force with 4 core targets (HIF1A, CASP1, PTGS2, and PPARG) among these 7 main targets. In addition, molecular dynamics simulation technology has further confirmed that the docked vitamin C molecules can well bind to the four core targets, and the stable complex structure is more conducive to the interaction between vitamin C and the targets to play the corresponding activity, which strongly supports the view that vitamin C may exert anti-inflammatory and antioxidant effects on HIE through these four targets.

HIF1A is a major regulator of cell adaptation to hypoxia. The activation of HIF1A can upregulate broad-spectrum target genes involved in glucose metabolism, angiogenesis, and erythropoiesis to avoid or minimize hypoxic brain damage [54, 55]. However, HIF1A promotes adaptation at low levels of hypoxia and is involved in promoting apoptosis at severe or prolonged hypoxia [56]. Many studies have confirmed that HIF1A is closely related to inflammation and the immune system. When immune cells are in a hypoxic state, activated HIF1A can regulate the survival and differentiation of immune cells such as Th17 cells through Th17 cell differentiation and other signal pathways, which has played an important role in controlling the expression of proinflammatory factors [57, 58]. In addition, the activation of HIF1A can induce stanniocalcin-1 in astrocytes to regulate glycolysis and reduce reactive oxygen species (ROS) levels to protect cells from hypoxia in an AMPK*α*1-dependent manner [59]. CASP1 is a key mediator of cell pyrolysis and participates in the inflammatory response and the development of various neurological diseases [60, 61]. According to reports, activation of CASP1 can induce astrocyte apoptosis after cerebral hypoxia [62]. In addition, inhibition of CASP1 activation reduced the cell viability loss of unbound bilirubinin-induced cortical astrocytes and the release of inflammatory cytokines such as IL-1*β* [63]. According to a study, inhibition of CASP1 can promote the cell proliferation in the subgranular and subventricular areas of the brain exposed to hyperoxia and reduce the degree of atrophy in these areas [64]. PTGS2 is related to neuroinflammatory activity. It was found that superoxide-mediated cell death was induced in primary cortical neurons [65]. According to reports, PTGS2 is involved in immune regulation and inflammation-related signaling pathways, such as the C-type lectin receptor signaling pathway, TNF signaling pathway, and IL-17 signaling pathway [66, 67]. In addition, PTGS2 is the target gene of HIF1A [68]. Related studies have shown that vitamin C can improve PTGS2-mediated related neuroinflammation and peripheral immune response and inflammation in serum [69]. As a stress-induced transcription factor, PPARG is involved in the regulation of anti-inflammatory mechanisms, oxidative stress, and neuronal death [70]. The activation of PPARG can improve neuronal apoptosis and ischemic brain damage via inhibiting the transcription of NF-*κ*B-driven NADPH oxidase regulatory subunits [71]. According to bioinformatics analysis, these four important targets may be involved in the damage regulation mechanism of HIE's inflammation, oxidative stress, and apoptosis.

In network pharmacology analysis, the effect of vitamin C in treating HIE is related to inflammation, immune response, and cell transcription regulation according to GO function annotation and KEGG pathway analysis. From the overall bioinformatics data, the effect of vitamin C on HIE is achieved by regulating the IL-17 signaling pathway, C-type lectin receptor signaling pathway, Th17 cell differentiation, B cell receptor signaling pathway, Nod-like receptor signaling pathway, tumor necrosis factor signaling pathway, T cell receptor signaling pathway, HIF-1 signaling pathway, and other signaling pathways, most of which are related to immunity and inflammation mediated by cerebral ischemia and hypoxia. For example, the dendritic cell-related C-type lectin-1 receptor signaling pathway can mediate neuroinflammation after ischemic stroke [72]. Nod-like receptor activation can increase the expression of caspase-1 and IL-1*β*, thus aggravating hypoxic-ischemic brain injury [73]. In addition, the HIF-1 signaling pathway can regulate downstream inflammation, oxidative stress, vascular endothelial factor, and metabolic-related signaling pathways among hypoxic-ischemic diseases [74]. Based on the KEGG Mapper database, the crosstalk between multiple signal pathways, such as the C-type lectin receptor signal pathway, can regulate downstream Th17 cell differentiation pathways, providing a further understanding of vitamin C in treating HIE. Therefore, it can be speculated that vitamin C-mediated anti-inflammatory and antioxidant effects help reduce the release of inflammatory cytokines in the brain and HIE-related immune inflammatory response.

Unfortunately, there are still some limitations of this study that this study only uses public databases and related software to predict related mechanisms, which may lead to uncertain results. Therefore, the research team intends to further verify these potential targets and signal pathways by means of animal experiments and cell experiments to clarify the potential pharmacological mechanism of vitamin C on HIE in future studies.

## 5. Conclusion

Overall, the emerging methods of network pharmacology, molecular docking, and molecular dynamics simulation were first used to systematically and intuitively reveal the mechanism and pharmacology of vitamin C in treating HIE through 4 targets (HIF1A, CASP1, PTGS2, and PPARG) working together with the IL-17 signal pathway, C-type lectin receptor signal pathway, HIF-1 signal pathway, and other immune and inflammation-related multisignal pathways, indicating that vitamin C may be one of the effective drugs for the treatment of HIE. Moreover, these core targets and signal pathways will contribute to the clinical practice of neonatal HIE in the future. In addition, the timing, dosage, and couse of application of vitamin C are also worthy of future research because of the different inflammatory reactions caused by HIE of different severities.

## Figures and Tables

**Table 1:** The potential targets of interaction between vitamin C and HIE.

UniProt ID	Gene	Protein
P08183	ABCB1	ATP-dependent translocase ABCB1
P09917	ALOX5	Polyunsaturated fatty acid 5-lipoxygenase
P10275	AR	Androgen receptor
P05023	ATP1A1	Sodium/potassium-transporting ATPase subunit alpha-1
P29466	CASP1	Caspase-1
P49841	GSK3B	Glycogen synthase kinase-3 beta
Q16665	HIF1A	Hypoxia-inducible factor 1-alpha
P11021	HSPA5	Endoplasmic reticulum chaperone BiP
P05412	JUN	Transcription factor AP-1
P03886	MT-ND1	NADH-ubiquinone oxidoreductase chain 1
P37231	PPARG	Peroxisome proliferator-activated receptor gamma
P17252	PRKCA	Protein kinase C alpha type
P35354	PTGS2	Prostaglandin G/H synthase 2
P40763	STAT3	Signal transducer and activator of transcription 3
P09488	GSTM1	Glutathione S-transferase mu 1
O14746	TERT	Telomerase reverse transcriptase

**Table 2:** Score results of molecular docking energy.

Target	PDB ID	Drug	Binding energy (kcal/mol)
CASP1	6F6R	Vitamin C	−6.5
GSK3B	1Q41	Vitamin C	−4.9
HIF1A	3HQR	Vitamin C	−6.8
JUN	2G01	Vitamin C	−4.7
PPARG	6T9C	vitaminC	−5.1
PTGS2	5F19	Vitamin C	−6.3
STAT3	5AX3	Vitamin C	−4.8

## Data Availability

The data used to support the findings of this study are included within the supplementary information file.

## Supplementary Materials

Supplementary Materials Supplementary Table 1: pathogenic targets of HIE. Supplementary Table 2: therapeutic targets of vitamin C. Supplementary Tables 3–5: results of biological processes, cell components, molecular functions, and KEGG pathway. Supplementary Figures 1–7: KEGG signaling pathway display diagram.
