# Supplementary material for: Mechanisms of Vitamin C Regulating Immune and Inflammation Associated with Neonatal Hypoxic-Ischemic Encephalopathy Based on Network Pharmacology and Molecular Simulation Technology
Source: Evid Based Complement Alternat Med. 2022 Feb 14;2022:4904325. doi: 10.1155/2022/4904325 (PMC8860524; doi:10.1155/2022/4904325)
Supplement: Supplementary Materials — Supplementary Table 1: pathogenic targets of HIE. Supplementary Table 2: therapeutic targets of vitamin C. Supplementary Tables 3–5: results of biological processes, cell components, molecular functions, and KEGG pathway. Supplementary Figures 1–7: KEGG signaling pathway display diagram. [file 4904325.f1.pdf]

Supplementary Table 1:Pathogenic targets of HIE from DisGeNET,Drugbank and Genecard databases

Gene	Gene	Gene	Gene	Gene	Gene	Gene	Gene	Gene	Gene	Gene
NOS2	CALR	CYP2E1	GPI	ITGB2	MIR27A	PDGFRB	SMAD3	IL6ST	MIR21	P2RX2
HIF1A	CANX	CYP3A4	GPT	ITM2B	MIR29A	PDP1	SMAD4	IMMT	MIR214	PANK2
VEGFA	CAPN1	DCAF8	GPX1	ITPR1	MIR29B1	PECAM1	SMARCA1	INA	MIR216A	PAPPA
IRAK1	CASP1	DCTN1	GPX3	JAG2	MIR30A	PEX1	SMARCA2	INHBA	MIR22	PARK7
IRAK4	CASP12	DCX	GRIA1	JAK1	MIR30B	PF4	SMARCA4	INS	MIR223	PARP1
EPO	CASP3	DDC	GRIN1	JUN	MIR30E	PFKM	SNHG3	INSR	MIR224	PC
S100B	CASP8	DDIT3	GRM1	KARS1	MIR34A	PGF	SOCS1	IRS1	MIR23A	PCDH19
MIR210	CASP9	DES	GRM5	KAT2B	MIR374A	PGK1	SOCS3	ITGA1	MIR23B	PCNA
AASS	CASR	DKK1	GRN	KCNB1	MIR483	PIGF	SOD1	ITGA4	MIR24-1	PDE5A
ABCB1	CAT	DLD	GSK3B	KCNH2	MIR485	PIK3C2A	SOD2	ITGAL	MIR25	PDGFB
ABCB11	CAV1	DLL4	GSR	KCNJ11	MIR503	PIK3CA	SORT1	ITGAM	MIR26A1	PDGFRA
ABCB7	CBS	DMD	GSS	KCNJ5	MIR93	PIK3CB	SOX5	SLC25A3	BDKRB2	CXCL10
ABCC1	CCDC88A	DMXL2	GSTM1	KCNMA1	MIRLET7B	PIK3CG	SP1	SLC2A1	BDNF	CXCL12
ABCG2	CCK	DNAH8	GSTP1	KDR	MKI67	PIK3R1	SPARC	SLC2A3	BECN1	CXCL8
ACADM	CCL11	DNASE1	GSTT1	KEAP1	MLYCD	PLA2G6	SPP1	SLC2A4	BGLAP	CXCR2
ACADSB	CCL18	DNM1	H19	KLF6	MME	PLAA	SPTAN1	SLC4A1	BIRC3	CXCR3
ACE	CCL2	DNM1L	HADHA	KLLN	MMP1	PLAT	SQSTM1	SLC6A3	BIRC5	CXCR4
ACE2	CCL26	DNMT3A	HADHB	KNG1	MMP14	PLAU	SRC	SLC6A4	BLVRB	CYC1
ACHE	CCL3	DOCK7	HAGLR	KRAS	MMP2	PLAUR	SST	SLC6A8	BMP6	CYCS
ACKR2	CCL4	DRD2	HBEGF	KRT18	MMP7	PLCG1	STAT1	SLC8A1	BNIP3	CYP11A1
ACTB	CCL5	DRD3	HBG2	KRT19	MMP9	PML	STAT3	SLC9A1	BRAF	CYP11B2
ACTC1	CCN1	DYNLL1	HCRT	KRT7	MOCS1	POLR1C	STMN1	SLPI	C3	CYP17A1
ACVR2A	CCN2	E2F1	HDAC1	KRT8	MOCS2	POMC	SUOX	WNT5A	CACNA1B	CYP19A1
ACVRL1	CCND1	E2F3	HDAC2	LAMA2	MOV10	PPARA	SYP	WT1	CALB1	CYP1A1
ADA	CCR1	ECE1	HDAC4	LBR	MPO	PPARG	TAC1	XBP1	CALCA	CYP2B6
ADAM10	CCR3	ECI2	HEY2	LCN2	MRE11	PPARGC1A	TCF4	XDH	ITGB1	CYP2C19
ADAM12	CCR5	EDN1	HGF	LDHA	MRPL12	PPIG	TCF7L2	XIAP	GDNF	IL10
ADAMTS13	CCR6	EDN3	HIGD1A	LEP	MSH2	PPP1R15A	TEAD1	YY1	GFAP	IL13
ADCY10	CD163	EDNRA	HJV	LEPR	MT-ATP6	PRDX1	TERC	ZEB1	GFER	IL15
ADCYAP1	CD34	EGF	HK1	LGALS3	MT-ATP8	PRKCA	TERT	SMAD2	GGT1	IL17A
ADIPOQ	CD36	EGFR	HLA-A	LIF	MT-CO2	PRKDC	TET2	MIR17	GH1	IL18

ADK	CD40	EIF2AK3	HLA-B	LOC10272405	MT-CO3	PRKN	TF	MIR181B1	GHR	IL18R1
ADM	CD44	EIF2AK4	HLA-DQB1	LONP1	MTDH	PRL	TFAM	MIR185	GHRL	IL1A
ADRB2	CD46	EIF2S1	HLA-G	LPL	MTHFR	PRNP	TFB1M	MIR191	GJA1	IL1B
AFG3L2	CD59	ELANE	HMGB1	LRP1	MT-ND1	PROM1	TFRC	MIR193A	GJB1	IL1RN
AFP	CDC42	EMD	HMOX1	LTF	MT-ND3	PSAP	TGFA	MIR195	GJB2	IL2
AGER	CDH1	ENG	HMOX2	MADD	MT-ND6	PSEN1	TGFB1	MIR199A1	GLS	IL3
AGT	CDH2	ENO1	HNF1A	MAG	MTOR	PTCD1	TGFB2	MIR200A	GLUD1	IL4
AGTR1	CDK1	ENO2	HNF4A	MALAT1	MTR	PTEN	TGFB3	MIR200B	GLUL	IL6
AGTR2	CDK2	ENPEP	HOTAIR	MAOA	MTRR	PTGDS	TGFBR1	MIR20A	GNAS	IL6R
AHSP	CDK4	EP300	HP	MAP1B	MT-TL1	PTGS2	TGFBR2	SLC12A2	GPC3	AXIN2
AIFM1	CDK5	EPRS1	HPSE	MAP2	MYC	PTK2	TH	SLC13A5	NTRK2	AZU1
AKR1B1	CDKN1A	ERBB2	HRAS	MAP2K1	NAGLU	PTK2B	THBD	SLC17A5	NTS	BACE1
AKT1	CDKN2A	ERCC6	HRH1	MAP2K4	NCAM1	PTPA	THPO	SLC1A1	NUP153	BAD
ALB	CDKN2B	ERN1	HSD11B2	MAPK1	NDE1	PTPN11	TIMP1	SLC1A2	OCLN	BAK1
ALDH2	CDKN3	ESR1	HSD17B10	MAPK10	NDUFA13	PVALB	TIMP2	SLC1A3	OLIG2	BAX
ALDH7A1	CEACAM1	ESRRG	HSP90AA1	MAPK14	NDUFA6	RAC1	TJP1	SLC1A4	OLR1	BCAP31
ALDH9A1	CES1	EZH2	HSPA1A	MAPK3	NDUFS4	RAF1	TKT	SLC1A6	OPRK1	BCKDK
ALOX5	CFI	EZR	HSPA4	MAPK8	NEAT1	RB1	TLR3	SLC22A5	OPRM1	BCL2
ALPP	CFLAR	F10	HSPA5	MAPT	NEFH	RBPJ	TLR4	SLC25A24	OXA1L	BCL2L1
ANGPT1	CFTR	F2	HSPA8	MB	NES	REN	TLR7	VCAM1	OXT	CSF2
ANGPT2	CGAS	F3	HSPB1	MBL2	NF1	REST	TMEM126B	VCL	GAMT	CSF3
ANK2	CGB3	F5	HSPD1	MBP	NFE2L2	RETN	TNF	VCP	GAPDH	CTH
ANK3	CHAT	F9	HTR1A	MCL1	NFIA	RHOA	TNFRSF1A	VIM	GAST	CTLA4
ANXA5	CHKA	FABP1	HTR3A	MDH1	NFKB1	RPS27A	TNFRSF1B	VIP	GATM	CTNNB1
AOX1	CHRNA7	FADD	HTRA1	MDK	NFKBIA	RPS6	TNIP1	VLDLR	GBA	CTSB
APAF1	CKB	FAS	HTRA2	MDM2	NGF	RPS6KB1	TNNT2	VWF	GC	CTSD
APC	CLU	FASLG	HTT	MEF2C	NISCH	RXRA	TOMM20	WARS1	GCDH	CTSG
APOE	COL4A1	FECH	ICAM1	MEG3	NLRP3	RYR1	TP53	WDR83	GCK	CUL3
APP	COMT	FGF13	ID1	MET	NLRX1	S100A1	TPH1	WNT1	GCLC	CXCL1
AQP1	COQ8B	FGF2	IDH1	MFF	NOS1	S100A12	TPI1	UTS2	GDF15	SIRT3
AQP4	COX4I1	FGFR1	IDH2	MFN2	NOS3	SAMHD1	TPP1	MIR143	NPY	SELPLG
AR	COX5A	FLNA	IDO1	MIR124-1	NOTCH1	SCN1A	TRAF6	MIR144	NQO1	SERPINA1
ARCN1	CP	FLT1	IFNA1	MIR125A	NOTCH2	SCN5A	TREX1	MIR145	NR1H4	SERPINA3

ARG1	CPOX	FMR1	IFNA2	MIR125B1	NOTCH3	SDHA	TRPC6	MIR146A	NR1I2	SERPINC1
ARG2	CPT1A	FN1	IFNB1	MIR126	NOTCH4	SDHB	TRPV1	MIR148A	NR2F2	SERPINE1
ARSA	CPT2	FOS	IFNG	MIR130A	NPPA	SELE	TRPV3	MIR148B	NR3C1	SFRP1
ASS1	CRAT	FOXM1	IGF1	MIR133A1	NPPB	SELL	TSC2	MIR150	NRAS	SGCB
ATF4	CREB1	FOXO1	IGF1R	MIR140	NPPC	SELP	TSPO	MIR155	NRP1	SGK1
ATF6	CREBBP	FOXP3	IGF2	ATP5F1A	CRP	G6PC1	TTN	MIR15A	NRP2	SHC1
ATP1A1	CRH	FST	IGF2BP2	ATP5F1E	CRYAB	G6PD	TTR	MIR15B	NSF	SIRT1
ATP2A2	CRHR1	FXN	IGF2R	ATXN2	CS	GAB1	TUBB3	MIR16-1	NTRK1	UCP2
IKBKG	IGFBP3	IGFBP2	IGFBP1	AVP	CSF1R	GAD1	TWIST1	UCHL1	UCN	UCP3
TYK2	UQCRFS1									

Supplementary Table 2:Therapeutic targets of vitamin C

Gene	Gene	Gene	Gene	Gene	Gene	Gene	Gene	Gene	Gene	Gene
VAV1	PTPN2	CXCR1	PDCD4	KDM5D	GSK3B	PAM	ALOX5	OGFOD1	DUSP3	EGLN2
TTL	PTPN1	CDC25B	KDM6B	PLOD1	FOLH1	P3H1	CASP1	OGFOD2	ABCB1	EGLN3
TRPV4	PTGS2	CA9	KDM5C	TMLHE	F2RL1	P3H2	HSPA5	ALKBH2	PTGS1	HIF1A
TERT	PRKCQ	CA2	KDM3A	P4HTM	EGLN2	P3H3	NDUFV1	ALKBH3	PTGS2	PGR
STAT3	PRKCH	CA1	KCNA3	EGLN1	EGLN1	P4HA1	MT-ND1	PRKCA	GABRA1	PDE4D
RPS6KA5	PRKCG	ATP6AP1	JUN	GRIA2	PRKCB	AR	PHYH	PRKCB	PRKCA	DBH
RORC	PRKCE	ATP2A1	IARS	DNA	PRKCA	ACP1	PLOD3			
LCT	PRKCD	ATP1A1	GSTM1	PLOD2	PPARG	NDUFA11	BBOX1			

Supplementary Table 3: GO Biological Processes

ID	Description	BgRatio	pvalue	p.adjust	geneID	Count
GO:1902895	positive regulation of pri-miRNA transcription by RNA polymerase II	40/18866	6.03E-10	7.34E-07	HIF1A/JUN/PPARG/STAT3	4
GO:1902893	regulation of pri-miRNA transcription by RNA polymerase II	51/18866	1.65E-09	7.82E-07	HIF1A/JUN/PPARG/STAT3	4
GO:0061614	pri-miRNA transcription by RNA polymerase II	53/18866	1.93E-09	7.82E-07	HIF1A/JUN/PPARG/STAT3	4
GO:0062013	positive regulation of small molecule metabolic process	154/18866	1.47E-07	3.96E-05	HIF1A/PPARG/PTGS2/STAT3	4
GO:0062012	regulation of small molecule metabolic process	456/18866	1.63E-07	3.96E-05	GSK3B/HIF1A/PPARG/PTGS2/STAT3	5
GO:0001936	regulation of endothelial cell proliferation	184/18866	3.00E-07	6.07E-05	HIF1A/JUN/PPARG/STAT3	4
GO:0001935	endothelial cell proliferation	199/18866	4.10E-07	7.12E-05	HIF1A/JUN/PPARG/STAT3	4
GO:0009612	response to mechanical stimulus	209/18866	4.99E-07	7.14E-05	CASP1/JUN/PPARG/PTGS2	4
GO:1901215	negative regulation of neuron death	212/18866	5.28E-07	7.14E-05	GSK3B/HIF1A/JUN/STAT3	4
GO:0033002	muscle cell proliferation	244/18866	9.27E-07	0.0001127	JUN/PPARG/PTGS2/STAT3	4
GO:0010632	regulation of epithelial cell migration	301/18866	2.14E-06	0.00023664	HIF1A/JUN/PPARG/PTGS2	4
GO:1901214	regulation of neuron death	321/18866	2.77E-06	0.00027506	GSK3B/HIF1A/JUN/STAT3	4
GO:0071496	cellular response to external stimulus	326/18866	2.94E-06	0.00027506	CASP1/JUN/PPARG/PTGS2	4
GO:0062197	cellular response to chemical stress	360/18866	4.36E-06	0.00031202	HIF1A/JUN/PPARG/PTGS2	4
GO:0070997	neuron death	360/18866	4.36E-06	0.00031202	GSK3B/HIF1A/JUN/STAT3	4
GO:0010631	epithelial cell migration	365/18866	4.61E-06	0.00031202	HIF1A/JUN/PPARG/PTGS2	4
GO:0090132	epithelium migration	368/18866	4.76E-06	0.00031202	HIF1A/JUN/PPARG/PTGS2	4
GO:0045862	positive regulation of proteolysis	370/18866	4.86E-06	0.00031202	CASP1/GSK3B/PPARG/STAT3	4
GO:0090130	tissue migration	374/18866	5.07E-06	0.00031202	HIF1A/JUN/PPARG/PTGS2	4

GO:0045639	positive regulation of myeloid cell differentiation	101/18866	5.13E-06	0.00031202	HIF1A/JUN/STAT3	3
GO:0050678	regulation of epithelial cell proliferation	395/18866	6.30E-06	0.00036075	HIF1A/JUN/PPARG/STAT3	4
GO:0002526	acute inflammatory response	111/18866	6.82E-06	0.00036075	PPARG/PTGS2/STAT3	3
GO:0045765	regulation of angiogenesis	403/18866	6.82E-06	0.00036075	HIF1A/PPARG/PTGS2/STAT3	4
GO:0001938	positive regulation of endothelial cell proliferation	113/18866	7.20E-06	0.00036461	HIF1A/JUN/STAT3	3
GO:0050727	regulation of inflammatory response	425/18866	8.42E-06	0.00040967	CASP1/PPARG/PTGS2/STAT3	4
GO:0030099	myeloid cell differentiation	431/18866	8.90E-06	0.00041213	HIF1A/JUN/PPARG/STAT3	4
GO:0052548	regulation of endopeptidase activity	434/18866	9.15E-06	0.00041213	CASP1/PPARG/PTGS2/STAT3	4
GO:1901342	regulation of vasculature development	444/18866	1.00E-05	0.00041686	HIF1A/PPARG/PTGS2/STAT3	4
GO:0001819	positive regulation of cytokine production	447/18866	1.03E-05	0.00041686	CASP1/HIF1A/PTGS2/STAT3	4
GO:0043434	response to peptide hormone	447/18866	1.03E-05	0.00041686	GSK3B/PPARG/PTGS2/STAT3	4
GO:0050673	epithelial cell proliferation	453/18866	1.08E-05	0.00042526	HIF1A/JUN/PPARG/STAT3	4
GO:0052547	regulation of peptidase activity	466/18866	1.21E-05	0.00046073	CASP1/PPARG/PTGS2/STAT3	4
GO:0001667	ameboidal-type cell migration	481/18866	1.37E-05	0.00050635	HIF1A/JUN/PPARG/PTGS2	4
GO:0007612	learning	145/18866	1.52E-05	0.00054424	HIF1A/JUN/PTGS2	3
GO:0001890	placenta development	156/18866	1.89E-05	0.00065818	HIF1A/PPARG/PTGS2	3
GO:0043467	regulation of generation of precursor metabolites and energy	162/18866	2.12E-05	0.00071521	GSK3B/HIF1A/STAT3	3
GO:0043535	regulation of blood vessel endothelial cell migration	164/18866	2.20E-05	0.00071521	HIF1A/PPARG/PTGS2	3
GO:0060965	negative regulation of gene silencing by miRNA	20/18866	2.24E-05	0.00071521	PPARG/STAT3	2
GO:0048660	regulation of smooth muscle cell proliferation	173/18866	2.58E-05	0.00080494	JUN/PPARG/PTGS2	3

GO:0048659	smooth muscle cell proliferation	175/18866	2.67E-05	0.00080605	JUN/PPARG/PTGS2	3
GO:0010634	positive regulation of epithelial cell migration	176/18866	2.72E-05	0.00080605	HIF1A/JUN/PTGS2	3
GO:0010950	positive regulation of endopeptidase activity	181/18866	2.96E-05	0.00082206	CASP1/PPARG/STAT3	3
GO:0060149	negative regulation of posttranscriptional gene silencing	23/18866	2.97E-05	0.00082206	PPARG/STAT3	2
GO:0060967	negative regulation of gene silencing by RNA	23/18866	2.97E-05	0.00082206	PPARG/STAT3	2
GO:0001959	regulation of cytokine-mediated signaling pathway	183/18866	3.05E-05	0.00082518	CASP1/HIF1A/PPARG	3
GO:0010869	regulation of receptor biosynthetic process	24/18866	3.24E-05	0.00085765	HIF1A/PPARG	2
GO:0043534	blood vessel endothelial cell migration	189/18866	3.36E-05	0.00086998	HIF1A/PPARG/PTGS2	3
GO:0060759	regulation of response to cytokine stimulus	196/18866	3.75E-05	0.00094953	CASP1/HIF1A/PPARG	3
GO:2000377	regulation of reactive oxygen species metabolic process	200/18866	3.98E-05	0.0009827	HIF1A/PTGS2/STAT3	3
GO:0010952	positive regulation of peptidase activity	201/18866	4.04E-05	0.0009827	CASP1/PPARG/STAT3	3
GO:1903708	positive regulation of hemopoiesis	204/18866	4.22E-05	0.00100696	HIF1A/JUN/STAT3	3
GO:0010575	positive regulation of vascular endothelial growth factor production	28/18866	4.44E-05	0.0010077	HIF1A/PTGS2	2
GO:0032800	receptor biosynthetic process	28/18866	4.44E-05	0.0010077	HIF1A/PPARG	2
GO:0045766	positive regulation of angiogenesis	208/18866	4.47E-05	0.0010077	HIF1A/PTGS2/STAT3	3
GO:0006109	regulation of carbohydrate metabolic process	210/18866	4.60E-05	0.00101409	GSK3B/HIF1A/STAT3	3

GO:0050679	positive regulation of epithelial cell proliferation	211/18866	4.67E-05	0.00101409	HIF1A/JUN/STAT3	3
GO:0043281	regulation of cysteine-type endopeptidase activity involved in apoptotic process	215/18866	4.94E-05	0.00105365	CASP1/PPARG/PTGS2	3
GO:0007623	circadian rhythm	218/18866	5.15E-05	0.00107912	GSK3B/JUN/PPARG	3
GO:0009266	response to temperature stimulus	233/18866	6.27E-05	0.00126239	GSK3B/PPARG/PTGS2	3
GO:2001234	negative regulation of apoptotic signaling pathway	233/18866	6.27E-05	0.00126239	GSK3B/HIF1A/PTGS2	3
GO:0071453	cellular response to oxygen levels	235/18866	6.44E-05	0.00126239	HIF1A/PPARG/PTGS2	3
GO:1904018	positive regulation of vasculature development	235/18866	6.44E-05	0.00126239	HIF1A/PTGS2/STAT3	3
GO:0045648	positive regulation of erythrocyte differentiation	34/18866	6.58E-05	0.00126613	HIF1A/STAT3	2
GO:0010594	regulation of endothelial cell migration	238/18866	6.68E-05	0.00126613	HIF1A/PPARG/PTGS2	3
GO:2000116	regulation of cysteine-type endopeptidase activity	239/18866	6.77E-05	0.00126613	CASP1/PPARG/PTGS2	3
GO:0042593	glucose homeostasis	245/18866	7.29E-05	0.0013215	HIF1A/PPARG/STAT3	3
GO:0033500	carbohydrate homeostasis	246/18866	7.37E-05	0.0013215	HIF1A/PPARG/STAT3	3
GO:0071634	regulation of transforming growth factor beta production	36/18866	7.39E-05	0.0013215	HIF1A/PTGS2	2
GO:0060969	negative regulation of gene silencing	37/18866	7.81E-05	0.00135458	PPARG/STAT3	2
GO:0071542	dopaminergic neuron differentiation	37/18866	7.81E-05	0.00135458	GSK3B/HIF1A	2
GO:0030224	monocyte differentiation	38/18866	8.24E-05	0.00135458	JUN/PPARG	2
GO:0045923	positive regulation of fatty acid metabolic process	38/18866	8.24E-05	0.00135458	PPARG/PTGS2	2
GO:0071604	transforming growth factor beta production	38/18866	8.24E-05	0.00135458	HIF1A/PTGS2	2

GO:1903131	mononuclear cell differentiation	38/18866	8.24E-05	0.00135458	JUN/PPARG	2
GO:0007611	learning or memory	260/18866	8.69E-05	0.00140941	HIF1A/JUN/PTGS2	3
GO:0045637	regulation of myeloid cell differentiation	263/18866	8.99E-05	0.00143907	HIF1A/JUN/STAT3	3
GO:0034504	protein localization to nucleus	277/18866	0.000104908	0.00165673	GSK3B/PTGS2/STAT3	3
GO:0043542	endothelial cell migration	286/18866	0.000115343	0.00178465	HIF1A/PPARG/PTGS2	3
GO:0010677	negative regulation of cellular carbohydrate metabolic process	45/18866	0.000115944	0.00178465	GSK3B/STAT3	2
GO:0072593	reactive oxygen species metabolic process	288/18866	0.00011775	0.00178981	HIF1A/PTGS2/STAT3	3
GO:0006953	acute-phase response	47/18866	0.000126556	0.00189991	PTGS2/STAT3	2
GO:0050890	cognition	302/18866	0.000135532	0.00191538	HIF1A/JUN/PTGS2	3
GO:0002673	regulation of acute inflammatory response	49/18866	0.000137629	0.00191538	PPARG/PTGS2	2
GO:0048511	rhythmic process	305/18866	0.000139557	0.00191538	GSK3B/JUN/PPARG	3
GO:0001961	positive regulation of cytokine-mediated signaling pathway	50/18866	0.000143339	0.00191538	CASP1/HIF1A	2
GO:0045646	regulation of erythrocyte differentiation	50/18866	0.000143339	0.00191538	HIF1A/STAT3	2
GO:0045981	positive regulation of nucleotide metabolic process	50/18866	0.000143339	0.00191538	HIF1A/STAT3	2
GO:0101023	vascular endothelial cell proliferation	50/18866	0.000143339	0.00191538	PPARG/STAT3	2
GO:1900544	positive regulation of purine nucleotide metabolic process	50/18866	0.000143339	0.00191538	HIF1A/STAT3	2
GO:1903580	positive regulation of ATP metabolic process	50/18866	0.000143339	0.00191538	HIF1A/STAT3	2
GO:1905562	regulation of vascular endothelial cell proliferation	50/18866	0.000143339	0.00191538	PPARG/STAT3	2

GO:0045912	negative regulation of carbohydrate metabolic process	51/18866	0.000149163	0.00197154	GSK3B/STAT3	2
GO:0007568	aging	319/18866	0.000159381	0.00208395	JUN/PTGS2/STAT3	3
GO:0032731	positive regulation of interleukin-1 beta production	53/18866	0.000161156	0.00208474	CASP1/STAT3	2
GO:0071375	cellular response to peptide hormone stimulus	330/18866	0.000176188	0.0022552	GSK3B/PPARG/STAT3	3
GO:0060760	positive regulation of response to cytokine stimulus	57/18866	0.000186519	0.00236257	CASP1/HIF1A	2
GO:0010506	regulation of autophagy	347/18866	0.000204376	0.00256207	GSK3B/HIF1A/STAT3	3
GO:0032732	positive regulation of interleukin-1 production	60/18866	0.000206744	0.00256531	CASP1/STAT3	2
GO:0010574	regulation of vascular endothelial growth factor production	61/18866	0.000213714	0.00261005	HIF1A/PTGS2	2
GO:0006913	nucleocytoplasmic transport	354/18866	0.000216789	0.00261005	GSK3B/PTGS2/STAT3	3
GO:0051222	positive regulation of protein transport	354/18866	0.000216789	0.00261005	GSK3B/HIF1A/PTGS2	3
GO:0051169	nuclear transport	357/18866	0.000222256	0.00264964	GSK3B/PTGS2/STAT3	3
GO:0002066	columnar/cuboidal epithelial cell development	64/18866	0.000235311	0.00277804	GSK3B/HIF1A	2
GO:0010038	response to metal ion	366/18866	0.000239196	0.00278462	HIF1A/JUN/PTGS2	3
GO:0051098	regulation of binding	367/18866	0.000241128	0.00278462	GSK3B/JUN/PPARG	3
GO:0010573	vascular endothelial growth factor production	65/18866	0.000242738	0.00278462	HIF1A/PTGS2	2
GO:1904951	positive regulation of establishment of protein localization	370/18866	0.000246987	0.00279211	GSK3B/HIF1A/PTGS2	3
GO:0046824	positive regulation of nucleocytoplasmic transport	66/18866	0.00025028	0.00279211	GSK3B/PTGS2	2
GO:2000378	negative regulation of reactive oxygen species metabolic process	66/18866	0.00025028	0.00279211	HIF1A/STAT3	2

GO:0001654	eye development	384/18866	0.000275558	0.00304617	HIF1A/JUN/STAT3	3
GO:0150063	visual system development	388/18866	0.000284099	0.00311229	HIF1A/JUN/STAT3	3
GO:0048880	sensory system development	394/18866	0.000297232	0.00320592	HIF1A/JUN/STAT3	3
GO:0045600	positive regulation of fat cell differentiation	72/18866	0.000297919	0.00320592	PPARG/PTGS2	2
GO:0070482	response to oxygen levels	396/18866	0.000301696	0.00320985	HIF1A/PPARG/PTGS2	3
GO:0042493	response to drug	397/18866	0.000303944	0.00320985	PPARG/PTGS2/STAT3	3
GO:1901653	cellular response to peptide	398/18866	0.000306203	0.00320985	GSK3B/PPARG/STAT3	3
GO:0046902	regulation of mitochondrial membrane permeability	77/18866	0.000340743	0.00348873	GSK3B/STAT3	2
GO:0150076	neuroinflammatory response	77/18866	0.000340743	0.00348873	JUN/PTGS2	2
GO:2001233	regulation of apoptotic signaling pathway	413/18866	0.000341413	0.00348873	GSK3B/HIF1A/PTGS2	3
GO:0043536	positive regulation of blood vessel endothelial cell migration	79/18866	0.000358666	0.00360445	HIF1A/PTGS2	2
GO:0071260	cellular response to mechanical stimulus	79/18866	0.000358666	0.00360445	CASP1/PTGS2	2
GO:0003151	outflow tract morphogenesis	80/18866	0.000367798	0.00363612	HIF1A/JUN	2
GO:0006110	regulation of glycolytic process	80/18866	0.000367798	0.00363612	HIF1A/STAT3	2
GO:0048145	regulation of fibroblast proliferation	83/18866	0.000395871	0.00388209	JUN/PPARG	2
GO:0048144	fibroblast proliferation	84/18866	0.000405455	0.00394426	JUN/PPARG	2
GO:0048608	reproductive structure development	443/18866	0.000419523	0.00404873	HIF1A/PPARG/PTGS2	3
GO:0061458	reproductive system development	447/18866	0.000430738	0.00409936	HIF1A/PPARG/PTGS2	3
GO:1904705	regulation of vascular associated smooth muscle cell proliferation	87/18866	0.000434883	0.00409936	JUN/PPARG	2
GO:1990874	vascular associated smooth muscle cell proliferation	87/18866	0.000434883	0.00409936	JUN/PPARG	2

GO:0090559	regulation of membrane permeability	88/18866	0.000444917	0.00414856	GSK3B/STAT3	2
GO:0050804	modulation of chemical synaptic transmission	454/18866	0.00045083	0.00414856	GSK3B/PTGS2/STAT3	3
GO:0051090	regulation of DNA-binding transcription factor activity	455/18866	0.000453749	0.00414856	JUN/PPARG/STAT3	3
GO:0099177	regulation of trans-synaptic signaling	455/18866	0.000453749	0.00414856	GSK3B/PTGS2/STAT3	3
GO:0006979	response to oxidative stress	458/18866	0.000462579	0.00416055	HIF1A/JUN/PTGS2	3
GO:0043470	regulation of carbohydrate catabolic process	90/18866	0.000465325	0.00416055	HIF1A/STAT3	2
GO:0070542	response to fatty acid	90/18866	0.000465325	0.00416055	PPARG/PTGS2	2
GO:0033273	response to vitamin	91/18866	0.000475697	0.00422225	PPARG/PTGS2	2
GO:0031667	response to nutrient levels	473/18866	0.000508408	0.00447988	JUN/PPARG/PTGS2	3
GO:0032651	regulation of interleukin-1 beta production	97/18866	0.000540292	0.00469282	CASP1/STAT3	2
GO:1901216	positive regulation of neuron death	97/18866	0.000540292	0.00469282	GSK3B/JUN	2
GO:2001243	negative regulation of intrinsic apoptotic signaling pathway	98/18866	0.00055145	0.00475577	HIF1A/PTGS2	2
GO:0019217	regulation of fatty acid metabolic process	101/18866	0.000585598	0.0050147	PPARG/PTGS2	2
GO:1903706	regulation of hemopoiesis	498/18866	0.000591156	0.00502689	HIF1A/JUN/STAT3	3
GO:0048661	positive regulation of smooth muscle cell proliferation	103/18866	0.000608922	0.00514201	JUN/PTGS2	2
GO:0032611	interleukin-1 beta production	106/18866	0.000644746	0.00540698	CASP1/STAT3	2
GO:1903426	regulation of reactive oxygen species biosynthetic process	107/18866	0.000656911	0.00547126	PTGS2/STAT3	2
GO:0043618	regulation of transcription from RNA polymerase II promoter in response to stress	109/18866	0.000681574	0.00563806	HIF1A/JUN	2

GO:0032652	regulation of interleukin-1 production	111/18866	0.000706683	0.00580626	CASP1/STAT3	2
GO:0046822	regulation of nucleocytoplasmic transport	112/18866	0.000719405	0.00587112	GSK3B/PTGS2	2
GO:0032963	collagen metabolic process	113/18866	0.000732237	0.005936	HIF1A/PPARG	2
GO:0006096	glycolytic process	115/18866	0.000758236	0.00606589	HIF1A/STAT3	2
GO:0043620	regulation of DNA-templated transcription in response to stress	115/18866	0.000758236	0.00606589	HIF1A/JUN	2
GO:0006757	ATP generation from ADP	116/18866	0.000771402	0.00609107	HIF1A/STAT3	2
GO:0060964	regulation of gene silencing by miRNA	116/18866	0.000771402	0.00609107	PPARG/STAT3	2
GO:0030282	bone mineralization	117/18866	0.000784678	0.00615593	HIF1A/PTGS2	2
GO:0021987	cerebral cortex development	118/18866	0.000798066	0.00622082	GSK3B/HIF1A	2
GO:0010822	positive regulation of mitochondrion organization	119/18866	0.000811564	0.00625825	GSK3B/HIF1A	2
GO:0060147	regulation of posttranscriptional gene silencing	120/18866	0.000825173	0.00625825	PPARG/STAT3	2
GO:0060966	regulation of gene silencing by RNA	120/18866	0.000825173	0.00625825	PPARG/STAT3	2
GO:0002065	columnar/cuboidal epithelial cell differentiation	121/18866	0.000838893	0.00625825	GSK3B/HIF1A	2
GO:0030218	erythrocyte differentiation	121/18866	0.000838893	0.00625825	HIF1A/STAT3	2
GO:0032612	interleukin-1 production	121/18866	0.000838893	0.00625825	CASP1/STAT3	2
GO:1900542	regulation of purine nucleotide metabolic process	121/18866	0.000838893	0.00625825	HIF1A/STAT3	2
GO:0042752	regulation of circadian rhythm	122/18866	0.000852724	0.00632264	GSK3B/PPARG	2
GO:0006140	regulation of nucleotide metabolic process	123/18866	0.000866665	0.00638706	HIF1A/STAT3	2
GO:0046031	ADP metabolic process	124/18866	0.000880717	0.00640102	HIF1A/STAT3	2
GO:0051101	regulation of DNA binding	124/18866	0.000880717	0.00640102	JUN/PPARG	2

GO:0008637	apoptotic mitochondrial changes	125/18866	0.000894879	0.00640102	GSK3B/JUN	2
GO:0034605	cellular response to heat	125/18866	0.000894879	0.00640102	GSK3B/PTGS2	2
GO:1903578	regulation of ATP metabolic process	125/18866	0.000894879	0.00640102	HIF1A/STAT3	2
GO:1900180	regulation of protein localization to nucleus	127/18866	0.000923534	0.00656735	GSK3B/PTGS2	2
GO:1903409	reactive oxygen species biosynthetic process	128/18866	0.000938027	0.00663163	PTGS2/STAT3	2
GO:0034101	erythrocyte homeostasis	129/18866	0.000952631	0.00669595	HIF1A/STAT3	2
GO:0010508	positive regulation of autophagy	131/18866	0.000982168	0.00686389	GSK3B/HIF1A	2
GO:0010595	positive regulation of endothelial cell migration	132/18866	0.000997101	0.00692843	HIF1A/PTGS2	2
GO:0006165	nucleoside diphosphate phosphorylation	133/18866	0.001012145	0.006993	HIF1A/STAT3	2
GO:0043280	positive regulation of cysteine-type endopeptidase activity involved in apoptotic process	134/18866	0.001027299	0.0070576	CASP1/PPARG	2
GO:0046939	nucleotide phosphorylation	135/18866	0.001042562	0.00712222	HIF1A/STAT3	2
GO:0009135	purine nucleoside diphosphate metabolic process	136/18866	0.001057935	0.00714694	HIF1A/STAT3	2
GO:0009179	purine ribonucleoside diphosphate metabolic process	136/18866	0.001057935	0.00714694	HIF1A/STAT3	2
GO:0009185	ribonucleoside diphosphate metabolic process	139/18866	0.001104713	0.00742172	HIF1A/STAT3	2
GO:0060968	regulation of gene silencing	140/18866	0.001120525	0.00748658	PPARG/STAT3	2
GO:0032355	response to estradiol	141/18866	0.001136446	0.00755147	PTGS2/STAT3	2
GO:0007006	mitochondrial membrane organization	142/18866	0.001152477	0.00761637	GSK3B/STAT3	2
GO:0045598	regulation of fat cell differentiation	143/18866	0.001168617	0.00768129	PPARG/PTGS2	2

GO:0010675	regulation of cellular carbohydrate metabolic process	148/18866	0.001250956	0.0081783	GSK3B/STAT3	2
GO:0014074	response to purine-containing compound	149/18866	0.001267752	0.00819993	PPARG/PTGS2	2
GO:0043524	negative regulation of neuron apoptotic process	149/18866	0.001267752	0.00819993	HIF1A/JUN	2
GO:0006606	protein import into nucleus	150/18866	0.001284656	0.0082653	PTGS2/STAT3	2
GO:2001056	positive regulation of cysteine-type endopeptidase activity	151/18866	0.001301669	0.00833068	CASP1/PPARG	2
GO:0001837	epithelial to mesenchymal transition	152/18866	0.001318791	0.00835234	GSK3B/HIF1A	2
GO:0006090	pyruvate metabolic process	152/18866	0.001318791	0.00835234	HIF1A/STAT3	2
GO:0045834	positive regulation of lipid metabolic process	153/18866	0.001336021	0.00841763	PPARG/PTGS2	2
GO:0002262	myeloid cell homeostasis	154/18866	0.001353361	0.00848292	HIF1A/STAT3	2
GO:0009132	nucleoside diphosphate metabolic process	155/18866	0.001370809	0.00854822	HIF1A/STAT3	2
GO:0048592	eye morphogenesis	156/18866	0.001388366	0.00861353	HIF1A/STAT3	2
GO:0050729	positive regulation of inflammatory response	158/18866	0.001423805	0.00878856	PTGS2/STAT3	2
GO:0031214	biomineral tissue development	163/18866	0.001514298	0.0092532	HIF1A/PTGS2	2
GO:0110148	biomineralization	163/18866	0.001514298	0.0092532	HIF1A/PTGS2	2
GO:0009408	response to heat	166/18866	0.001569893	0.00949746	GSK3B/PTGS2	2
GO:2001242	regulation of intrinsic apoptotic signaling pathway	166/18866	0.001569893	0.00949746	HIF1A/PTGS2	2
GO:0051100	negative regulation of binding	169/18866	0.001626459	0.00979096	GSK3B/JUN	2
GO:0007584	response to nutrient	171/18866	0.001664708	0.00992297	PPARG/PTGS2	2
GO:0051170	import into nucleus	171/18866	0.001664708	0.00992297	PTGS2/STAT3	2
GO:0050806	positive regulation of synaptic transmission	172/18866	0.001683994	0.00998896	GSK3B/PTGS2	2
GO:0021543	pallium development	173/18866	0.001703388	0.01005495	GSK3B/HIF1A	2
GO:0001659	temperature homeostasis	174/18866	0.00172289	0.01007228	PTGS2/STAT3	2

GO:0051099	positive regulation of binding	174/18866	0.00172289	0.01007228	GSK3B/PPARG	2
GO:0048469	cell maturation	179/18866	0.001822008	0.01060077	HIF1A/PPARG	2
GO:0071346	cellular response to interferon-gamma	182/18866	0.001882766	0.01085044	CASP1/PPARG	2
GO:0090316	positive regulation of intracellular protein transport	182/18866	0.001882766	0.01085044	GSK3B/PTGS2	2
GO:0008217	regulation of blood pressure	187/18866	0.001986167	0.01139236	PPARG/PTGS2	2
GO:0010565	regulation of cellular ketone metabolic process	189/18866	0.002028276	0.01157926	PPARG/PTGS2	2
GO:0010821	regulation of mitochondrion organization	191/18866	0.00207081	0.01171212	GSK3B/HIF1A	2
GO:0048167	regulation of synaptic plasticity	191/18866	0.00207081	0.01171212	GSK3B/PTGS2	2
GO:0071248	cellular response to metal ion	193/18866	0.002113771	0.01189975	JUN/PTGS2	2
GO:0043112	receptor metabolic process	198/18866	0.002223032	0.01245717	HIF1A/PPARG	2
GO:0016052	carbohydrate catabolic process	200/18866	0.002267479	0.0125902	HIF1A/STAT3	2
GO:0017038	protein import	200/18866	0.002267479	0.0125902	PTGS2/STAT3	2
GO:0031099	regeneration	201/18866	0.002289862	0.01265669	JUN/PPARG	2
GO:0009746	response to hexose	202/18866	0.00231235	0.01266585	HIF1A/PTGS2	2
GO:0034341	response to interferon-gamma	202/18866	0.00231235	0.01266585	CASP1/PPARG	2
GO:0042594	response to starvation	206/18866	0.002403362	0.01310533	JUN/PPARG	2
GO:0034284	response to monosaccharide	207/18866	0.002426379	0.01317177	HIF1A/PTGS2	2
GO:0071456	cellular response to hypoxia	208/18866	0.002449502	0.0132382	HIF1A/PTGS2	2
GO:0002573	myeloid leukocyte differentiation	210/18866	0.002496063	0.01343015	JUN/PPARG	2
GO:0043523	regulation of neuron apoptotic process	214/18866	0.002590451	0.0138766	HIF1A/JUN	2
GO:0036294	cellular response to decreased oxygen levels	218/18866	0.00268652	0.01432811	HIF1A/PTGS2	2
GO:0002064	epithelial cell development	221/18866	0.002759673	0.01452711	GSK3B/HIF1A	2
GO:0031669	cellular response to nutrient levels	221/18866	0.002759673	0.01452711	JUN/PPARG	2

GO:0071241	cellular response to inorganic substance	221/18866	0.002759673	0.01452711	JUN/PTGS2	2
GO:0032388	positive regulation of intracellular transport	222/18866	0.002784267	0.0145934	GSK3B/PTGS2	2
GO:0032869	cellular response to insulin stimulus	226/18866	0.002883689	0.01504964	GSK3B/PPARG	2
GO:0048762	mesenchymal cell differentiation	229/18866	0.002959352	0.01537851	GSK3B/HIF1A	2
GO:0010001	glial cell differentiation	230/18866	0.002984782	0.01544466	PPARG/STAT3	2
GO:0009743	response to carbohydrate	233/18866	0.003061695	0.01577552	HIF1A/PTGS2	2
GO:0097305	response to alcohol	234/18866	0.003087541	0.01584103	PPARG/STAT3	2
GO:0045444	fat cell differentiation	235/18866	0.003113492	0.01584103	PPARG/PTGS2	2
GO:0071695	anatomical structure maturation	235/18866	0.003113492	0.01584103	HIF1A/PPARG	2
GO:0046777	protein autophosphorylation	237/18866	0.003165703	0.01603956	GSK3B/JUN	2
GO:0051402	neuron apoptotic process	245/18866	0.003378697	0.0170477	HIF1A/JUN	2
GO:0071216	cellular response to biotic stimulus	246/18866	0.003405786	0.01711337	CASP1/GSK3B	2
GO:0006352	DNA-templated transcription, initiation	249/18866	0.003487675	0.01745273	JUN/PPARG	2
GO:0031668	cellular response to extracellular stimulus	253/18866	0.003598303	0.01759909	JUN/PPARG	2
GO:0045926	negative regulation of growth	254/18866	0.003626217	0.01759909	HIF1A/PPARG	2
GO:0048872	homeostasis of number of cells	256/18866	0.003682354	0.01759909	HIF1A/STAT3	2
GO:0001660	fever generation	10/18866	0.003705072	0.01759909	PTGS2	1
GO:0010887	negative regulation of cholesterol storage	10/18866	0.003705072	0.01759909	PPARG	1
GO:0042541	hemoglobin biosynthetic process	10/18866	0.003705072	0.01759909	HIF1A	1
GO:0045713	low-density lipoprotein particle receptor biosynthetic process	10/18866	0.003705072	0.01759909	PPARG	1
GO:0051541	elastin metabolic process	10/18866	0.003705072	0.01759909	HIF1A	1

GO:0070099	regulation of chemokine-mediated signaling pathway	10/18866	0.003705072	0.01759909	HIF1A	1
GO:0071104	response to interleukin-9	10/18866	0.003705072	0.01759909	STAT3	1
GO:1904338	regulation of dopaminergic neuron differentiation	10/18866	0.003705072	0.01759909	GSK3B	1
GO:1904779	regulation of protein localization to centrosome	10/18866	0.003705072	0.01759909	GSK3B	1
GO:2001054	negative regulation of mesenchymal cell apoptotic process	10/18866	0.003705072	0.01759909	HIF1A	1
GO:0003007	heart morphogenesis	258/18866	0.003738902	0.01762246	HIF1A/JUN	2
GO:0021537	telencephalon development	259/18866	0.00376733	0.01762246	GSK3B/HIF1A	2
GO:0042180	cellular ketone metabolic process	260/18866	0.00379586	0.01762246	PPARG/PTGS2	2
GO:0033157	regulation of intracellular protein transport	264/18866	0.003911005	0.01762246	GSK3B/PTGS2	2
GO:0048863	stem cell differentiation	264/18866	0.003911005	0.01762246	HIF1A/STAT3	2
GO:0030522	intracellular receptor signaling pathway	265/18866	0.003940047	0.01762246	PPARG/STAT3	2
GO:0009165	nucleotide biosynthetic process	266/18866	0.003969191	0.01762246	PTGS2/STAT3	2
GO:0090596	sensory organ morphogenesis	269/18866	0.004057236	0.01762246	HIF1A/STAT3	2
GO:1901293	nucleoside phosphate biosynthetic process	269/18866	0.004057236	0.01762246	PTGS2/STAT3	2
GO:0019371	cyclooxygenase pathway	11/18866	0.004074931	0.01762246	PTGS2	1
GO:0031652	positive regulation of heat generation	11/18866	0.004074931	0.01762246	PTGS2	1
GO:0031915	positive regulation of synaptic plasticity	11/18866	0.004074931	0.01762246	PTGS2	1
GO:0033197	response to vitamin E	11/18866	0.004074931	0.01762246	PPARG	1
GO:0033210	leptin-mediated signaling pathway	11/18866	0.004074931	0.01762246	STAT3	1
GO:0033483	gas homeostasis	11/18866	0.004074931	0.01762246	HIF1A	1

GO:0042789	mRNA transcription by RNA polymerase II	11/18866	0.004074931	0.01762246	STAT3	1
GO:0043619	regulation of transcription from RNA polymerase II promoter in response to oxidative stress	11/18866	0.004074931	0.01762246	HIF1A	1
GO:0045657	positive regulation of monocyte differentiation	11/18866	0.004074931	0.01762246	JUN	1
GO:0051974	negative regulation of telomerase activity	11/18866	0.004074931	0.01762246	PPARG	1
GO:0070106	interleukin-27-mediated signaling pathway	11/18866	0.004074931	0.01762246	STAT3	1
GO:0070757	interleukin-35-mediated signaling pathway	11/18866	0.004074931	0.01762246	STAT3	1
GO:0090269	fibroblast growth factor production	11/18866	0.004074931	0.01762246	PTGS2	1
GO:0090270	regulation of fibroblast growth factor production	11/18866	0.004074931	0.01762246	PTGS2	1
GO:0106049	regulation of cellular response to osmotic stress	11/18866	0.004074931	0.01762246	PTGS2	1
GO:1903265	positive regulation of tumor necrosis factor-mediated signaling pathway	11/18866	0.004074931	0.01762246	CASP1	1
GO:0051091	positive regulation of DNA-binding transcription factor activity	270/18866	0.004086788	0.01762246	PPARG/STAT3	2
GO:0006839	mitochondrial transport	271/18866	0.004116442	0.01768761	GSK3B/STAT3	2
GO:0015980	energy derivation by oxidation of organic compounds	278/18866	0.004326869	0.01838341	GSK3B/HIF1A	2
GO:0045165	cell fate commitment	278/18866	0.004326869	0.01838341	PPARG/STAT3	2
GO:0006983	ER overload response	12/18866	0.004444672	0.01838341	GSK3B	1
GO:0031392	regulation of prostaglandin biosynthetic process	12/18866	0.004444672	0.01838341	PTGS2	1

GO:0060019	radial glial cell differentiation	12/18866	0.004444672	0.01838341	STAT3	1
GO:0070243	regulation of thymocyte apoptotic process	12/18866	0.004444672	0.01838341	HIF1A	1
GO:0072540	T-helper 17 cell lineage commitment	12/18866	0.004444672	0.01838341	STAT3	1
GO:0097201	negative regulation of transcription from RNA polymerase II promoter in response to stress	12/18866	0.004444672	0.01838341	JUN	1
GO:1905461	positive regulation of vascular associated smooth muscle cell apoptotic process	12/18866	0.004444672	0.01838341	PPARG	1
GO:2001053	regulation of mesenchymal cell apoptotic process	12/18866	0.004444672	0.01838341	HIF1A	1
GO:2001223	negative regulation of neuron migration	12/18866	0.004444672	0.01838341	STAT3	1
GO:0032868	response to insulin	283/18866	0.004480218	0.01846761	GSK3B/PPARG	2
GO:0021700	developmental maturation	287/18866	0.004604718	0.01869106	HIF1A/PPARG	2
GO:0060485	mesenchyme development	290/18866	0.004699152	0.01869106	GSK3B/HIF1A	2
GO:0097193	intrinsic apoptotic signaling pathway	290/18866	0.004699152	0.01869106	HIF1A/PTGS2	2
GO:0002674	negative regulation of acute inflammatory response	13/18866	0.004814296	0.01869106	PPARG	1
GO:0010745	negative regulation of macrophage derived foam cell differentiation	13/18866	0.004814296	0.01869106	PPARG	1
GO:0010870	positive regulation of receptor biosynthetic process	13/18866	0.004814296	0.01869106	HIF1A	1
GO:0014842	regulation of skeletal muscle satellite cell proliferation	13/18866	0.004814296	0.01869106	STAT3	1
GO:0031053	primary miRNA processing	13/18866	0.004814296	0.01869106	STAT3	1
GO:0031650	regulation of heat generation	13/18866	0.004814296	0.01869106	PTGS2	1

GO:0031953	negative regulation of protein autophosphorylation	13/18866	0.004814296	0.01869106	JUN	1
GO:0032352	positive regulation of hormone metabolic process	13/18866	0.004814296	0.01869106	HIF1A	1
GO:0035723	interleukin-15-mediated signaling pathway	13/18866	0.004814296	0.01869106	STAT3	1
GO:0060100	positive regulation of phagocytosis, engulfment	13/18866	0.004814296	0.01869106	PPARG	1
GO:0061029	eyelid development in camera-type eye	13/18866	0.004814296	0.01869106	JUN	1
GO:0071350	cellular response to interleukin-15	13/18866	0.004814296	0.01869106	STAT3	1
GO:0097152	mesenchymal cell apoptotic process	13/18866	0.004814296	0.01869106	HIF1A	1
GO:1905155	positive regulation of membrane invagination	13/18866	0.004814296	0.01869106	PPARG	1
GO:0034976	response to endoplasmic reticulum stress	294/18866	0.004826475	0.01869106	GSK3B/JUN	2
GO:0044262	cellular carbohydrate metabolic process	294/18866	0.004826475	0.01869106	GSK3B/STAT3	2
GO:0050768	negative regulation of neurogenesis	295/18866	0.004858557	0.01875557	GSK3B/STAT3	2
GO:0051348	negative regulation of transferase activity	296/18866	0.00489074	0.01882006	GSK3B/PPARG	2
GO:0010889	regulation of sequestering of triglyceride	14/18866	0.005183802	0.01910153	PPARG	1
GO:0014841	skeletal muscle satellite cell proliferation	14/18866	0.005183802	0.01910153	STAT3	1
GO:0014857	regulation of skeletal muscle cell proliferation	14/18866	0.005183802	0.01910153	STAT3	1
GO:0019896	axonal transport of mitochondrion	14/18866	0.005183802	0.01910153	HIF1A	1

GO:0034356	NAD biosynthesis via nicotinamide riboside salvage pathway	14/18866	0.005183802	0.01910153	PTGS2	1
GO:0036295	cellular response to increased oxygen levels	14/18866	0.005183802	0.01910153	PPARG	1
GO:0043922	negative regulation by host of viral transcription	14/18866	0.005183802	0.01910153	JUN	1
GO:0046321	positive regulation of fatty acid oxidation	14/18866	0.005183802	0.01910153	PPARG	1
GO:0047484	regulation of response to osmotic stress	14/18866	0.005183802	0.01910153	PTGS2	1
GO:0050872	white fat cell differentiation	14/18866	0.005183802	0.01910153	PPARG	1
GO:0060397	growth hormone receptor signaling pathway via JAK-STAT	14/18866	0.005183802	0.01910153	STAT3	1
GO:0070672	response to interleukin-15	14/18866	0.005183802	0.01910153	STAT3	1
GO:1903799	negative regulation of production of miRNAs involved in gene silencing by miRNA	14/18866	0.005183802	0.01910153	STAT3	1
GO:2001279	regulation of unsaturated fatty acid biosynthetic process	14/18866	0.005183802	0.01910153	PTGS2	1
GO:0042063	gliogenesis	307/18866	0.005251363	0.01929202	PPARG/STAT3	2
GO:0018105	peptidyl-serine phosphorylation	310/18866	0.005351813	0.01954296	GSK3B/PTGS2	2
GO:0034599	cellular response to oxidative stress	310/18866	0.005351813	0.01954296	HIF1A/JUN	2
GO:0046034	ATP metabolic process	311/18866	0.005385496	0.01960707	HIF1A/STAT3	2
GO:0006089	lactate metabolic process	15/18866	0.005553191	0.01968712	HIF1A	1
GO:0014856	skeletal muscle cell proliferation	15/18866	0.005553191	0.01968712	STAT3	1
GO:0020027	hemoglobin metabolic process	15/18866	0.005553191	0.01968712	HIF1A	1
GO:0045820	negative regulation of glycolytic process	15/18866	0.005553191	0.01968712	STAT3	1

GO:0060099	regulation of phagocytosis, engulfment	15/18866	0.005553191	0.01968712	PPARG	1
GO:0070885	negative regulation of calcineurin-NFAT signaling cascade	15/18866	0.005553191	0.01968712	GSK3B	1
GO:0090594	inflammatory response to wounding	15/18866	0.005553191	0.01968712	HIF1A	1
GO:0106057	negative regulation of calcineurin-mediated signaling	15/18866	0.005553191	0.01968712	GSK3B	1
GO:2001171	positive regulation of ATP biosynthetic process	15/18866	0.005553191	0.01968712	STAT3	1
GO:0009416	response to light stimulus	319/18866	0.005658536	0.01994429	HIF1A/PTGS2	2
GO:0051961	negative regulation of nervous system development	319/18866	0.005658536	0.01994429	GSK3B/STAT3	2
GO:0034612	response to tumor necrosis factor	320/18866	0.005693112	0.02000816	CASP1/PTGS2	2
GO:0002070	epithelial cell maturation	16/18866	0.005922462	0.02028652	HIF1A	1
GO:0002295	T-helper cell lineage commitment	16/18866	0.005922462	0.02028652	STAT3	1
GO:0019372	lipoxygenase pathway	16/18866	0.005922462	0.02028652	PTGS2	1
GO:0032225	regulation of synaptic transmission, dopaminergic	16/18866	0.005922462	0.02028652	PTGS2	1
GO:0060576	intestinal epithelial cell development	16/18866	0.005922462	0.02028652	HIF1A	1
GO:0060850	regulation of transcription involved in cell fate commitment	16/18866	0.005922462	0.02028652	PPARG	1
GO:0071380	cellular response to prostaglandin E stimulus	16/18866	0.005922462	0.02028652	PPARG	1
GO:1905050	positive regulation of metallopeptidase activity	16/18866	0.005922462	0.02028652	STAT3	1
GO:1905153	regulation of membrane invagination	16/18866	0.005922462	0.02028652	PPARG	1

GO:0071214	cellular response to abiotic stimulus	331/18866	0.006079979	0.020511	CASP1/PTGS2	2
GO:0104004	cellular response to environmental stimulus	331/18866	0.006079979	0.020511	CASP1/PTGS2	2
GO:0043010	camera-type eye development	332/18866	0.00611574	0.020511	HIF1A/JUN	2
GO:0018209	peptidyl-serine modification	333/18866	0.0061516	0.020511	GSK3B/PTGS2	2
GO:0032496	response to lipopolysaccharide	334/18866	0.006187558	0.020511	CASP1/PTGS2	2
GO:0009299	mRNA transcription	17/18866	0.006291616	0.020511	STAT3	1
GO:0030730	sequestering of triglyceride	17/18866	0.006291616	0.020511	PPARG	1
GO:0030949	positive regulation of vascular endothelial growth factor receptor signaling pathway	17/18866	0.006291616	0.020511	HIF1A	1
GO:0031000	response to caffeine	17/18866	0.006291616	0.020511	PPARG	1
GO:0031649	heat generation	17/18866	0.006291616	0.020511	PTGS2	1
GO:0032966	negative regulation of collagen biosynthetic process	17/18866	0.006291616	0.020511	PPARG	1
GO:0042953	lipoprotein transport	17/18866	0.006291616	0.020511	PPARG	1
GO:0043923	positive regulation by host of viral transcription	17/18866	0.006291616	0.020511	JUN	1
GO:0045986	negative regulation of smooth muscle contraction	17/18866	0.006291616	0.020511	PTGS2	1
GO:0050665	hydrogen peroxide biosynthetic process	17/18866	0.006291616	0.020511	STAT3	1
GO:0060644	mammary gland epithelial cell differentiation	17/18866	0.006291616	0.020511	HIF1A	1
GO:0070242	thymocyte apoptotic process	17/18866	0.006291616	0.020511	HIF1A	1
GO:0090336	positive regulation of brown fat cell differentiation	17/18866	0.006291616	0.020511	PTGS2	1
GO:1903829	positive regulation of cellular protein localization	338/18866	0.006332372	0.02058867	GSK3B/PTGS2	2
GO:0010721	negative regulation of cell development	343/18866	0.006515594	0.0211279	GSK3B/STAT3	2

GO:0010713	negative regulation of collagen metabolic process	18/18866	0.006660651	0.02120249	PPARG	1
GO:0010885	regulation of cholesterol storage	18/18866	0.006660651	0.02120249	PPARG	1
GO:0035994	response to muscle stretch	18/18866	0.006660651	0.02120249	JUN	1
GO:0044872	lipoprotein localization	18/18866	0.006660651	0.02120249	PPARG	1
GO:0046885	regulation of hormone biosynthetic process	18/18866	0.006660651	0.02120249	HIF1A	1
GO:0061298	retina vasculature development in camera-type eye	18/18866	0.006660651	0.02120249	HIF1A	1
GO:0071318	cellular response to ATP	18/18866	0.006660651	0.02120249	PTGS2	1
GO:0030336	negative regulation of cell migration	350/18866	0.006776209	0.02151402	PPARG/STAT3	2
GO:0048638	regulation of developmental growth	353/18866	0.006889363	0.02153138	GSK3B/STAT3	2
GO:0002237	response to molecule of bacterial origin	356/18866	0.007003391	0.02153138	CASP1/PTGS2	2
GO:0030011	maintenance of cell polarity	19/18866	0.00702957	0.02153138	GSK3B	1
GO:0033189	response to vitamin A	19/18866	0.00702957	0.02153138	PPARG	1
GO:0034393	positive regulation of smooth muscle cell apoptotic process	19/18866	0.00702957	0.02153138	PPARG	1
GO:0043373	CD4-positive, alpha-beta T cell lineage commitment	19/18866	0.00702957	0.02153138	STAT3	1
GO:0044320	cellular response to leptin stimulus	19/18866	0.00702957	0.02153138	STAT3	1
GO:0060749	mammary gland alveolus development	19/18866	0.00702957	0.02153138	HIF1A	1
GO:0061377	mammary gland lobule development	19/18866	0.00702957	0.02153138	HIF1A	1
GO:0070233	negative regulation of T cell apoptotic process	19/18866	0.00702957	0.02153138	HIF1A	1

GO:1902176	negative regulation of oxidative stress-induced intrinsic apoptotic signaling pathway	19/18866	0.00702957	0.02153138	HIF1A	1
GO:1904683	regulation of metalloendopeptidase activity	19/18866	0.00702957	0.02153138	STAT3	1
GO:1905288	vascular associated smooth muscle cell apoptotic process	19/18866	0.00702957	0.02153138	PPARG	1
GO:1905459	regulation of vascular associated smooth muscle cell apoptotic process	19/18866	0.00702957	0.02153138	PPARG	1
GO:0032386	regulation of intracellular transport	358/18866	0.007079896	0.02163104	GSK3B/PTGS2	2
GO:0001666	response to hypoxia	359/18866	0.007118293	0.02169385	HIF1A/PTGS2	2
GO:2000146	negative regulation of cell motility	365/18866	0.00735071	0.02215867	PPARG/STAT3	2
GO:0003323	type B pancreatic cell development	20/18866	0.007398371	0.02215867	GSK3B	1
GO:0010042	response to manganese ion	20/18866	0.007398371	0.02215867	PTGS2	1
GO:0010878	cholesterol storage	20/18866	0.007398371	0.02215867	PPARG	1
GO:0071636	positive regulation of transforming growth factor beta production	20/18866	0.007398371	0.02215867	PTGS2	1
GO:0097709	connective tissue replacement	20/18866	0.007398371	0.02215867	HIF1A	1
GO:2001169	regulation of ATP biosynthetic process	20/18866	0.007398371	0.02215867	STAT3	1
GO:0036293	response to decreased oxygen levels	371/18866	0.007586601	0.02243406	HIF1A/PTGS2	2
GO:0002363	alpha-beta T cell lineage commitment	21/18866	0.007767055	0.02243406	STAT3	1
GO:0010310	regulation of hydrogen peroxide metabolic process	21/18866	0.007767055	0.02243406	STAT3	1
GO:0016540	protein autoprocessing	21/18866	0.007767055	0.02243406	CASP1	1

GO:0030502	negative regulation of bone mineralization	21/18866	0.007767055	0.02243406	HIF1A	1
GO:0030728	ovulation	21/18866	0.007767055	0.02243406	PTGS2	1
GO:0035357	peroxisome proliferator activated receptor signaling pathway	21/18866	0.007767055	0.02243406	PPARG	1
GO:0046716	muscle cell cellular homeostasis	21/18866	0.007767055	0.02243406	HIF1A	1
GO:0046827	positive regulation of protein export from nucleus	21/18866	0.007767055	0.02243406	GSK3B	1
GO:0071498	cellular response to fluid shear stress	21/18866	0.007767055	0.02243406	PTGS2	1
GO:1900543	negative regulation of purine nucleotide metabolic process	21/18866	0.007767055	0.02243406	STAT3	1
GO:1903204	negative regulation of oxidative stress-induced neuron death	21/18866	0.007767055	0.02243406	HIF1A	1
GO:1903599	positive regulation of autophagy of mitochondrion	21/18866	0.007767055	0.02243406	HIF1A	1
GO:1904886	beta-catenin destruction complex disassembly	21/18866	0.007767055	0.02243406	GSK3B	1
GO:2000737	negative regulation of stem cell differentiation	21/18866	0.007767055	0.02243406	STAT3	1
GO:0010888	negative regulation of lipid storage	22/18866	0.008135621	0.02310247	PPARG	1
GO:0043369	CD4-positive or CD8-positive, alpha-beta T cell lineage commitment	22/18866	0.008135621	0.02310247	STAT3	1
GO:0045655	regulation of monocyte differentiation	22/18866	0.008135621	0.02310247	JUN	1
GO:0045980	negative regulation of nucleotide metabolic process	22/18866	0.008135621	0.02310247	STAT3	1

GO:0051000	positive regulation of nitric-oxide synthase activity	22/18866	0.008135621	0.02310247	HIF1A	1
GO:0055093	response to hyperoxia	22/18866	0.008135621	0.02310247	PPARG	1
GO:0071379	cellular response to prostaglandin stimulus	22/18866	0.008135621	0.02310247	PPARG	1
GO:0031349	positive regulation of defense response	385/18866	0.00815046	0.02310247	PTGS2/STAT3	2
GO:0031331	positive regulation of cellular catabolic process	390/18866	0.008356377	0.02323809	GSK3B/HIF1A	2
GO:0030900	forebrain development	391/18866	0.008397847	0.02323809	GSK3B/HIF1A	2
GO:0002052	positive regulation of neuroblast proliferation	23/18866	0.00850407	0.02323809	HIF1A	1
GO:0010226	response to lithium ion	23/18866	0.00850407	0.02323809	PTGS2	1
GO:0010288	response to lead ion	23/18866	0.00850407	0.02323809	PTGS2	1
GO:0032799	low-density lipoprotein receptor particle metabolic process	23/18866	0.00850407	0.02323809	PPARG	1
GO:0034643	establishment of mitochondrion localization, microtubule-mediated	23/18866	0.00850407	0.02323809	HIF1A	1
GO:0035162	embryonic hemopoiesis	23/18866	0.00850407	0.02323809	HIF1A	1
GO:0045723	positive regulation of fatty acid biosynthetic process	23/18866	0.00850407	0.02323809	PTGS2	1
GO:0047497	mitochondrion transport along microtubule	23/18866	0.00850407	0.02323809	HIF1A	1
GO:0048714	positive regulation of oligodendrocyte differentiation	23/18866	0.00850407	0.02323809	PPARG	1
GO:0071404	cellular response to low-density lipoprotein particle stimulus	23/18866	0.00850407	0.02323809	PPARG	1

GO:1900017	positive regulation of cytokine production involved in inflammatory response	23/18866	0.00850407	0.02323809	STAT3	1
GO:1901522	positive regulation of transcription from RNA polymerase II promoter involved in cellular response to chemical stimulus	23/18866	0.00850407	0.02323809	HIF1A	1
GO:1901984	negative regulation of protein acetylation	23/18866	0.00850407	0.02323809	GSK3B	1
GO:1903798	regulation of production of miRNAs involved in gene silencing by miRNA	23/18866	0.00850407	0.02323809	STAT3	1
GO:0006631	fatty acid metabolic process	396/18866	0.008606618	0.02346558	PPARG/PTGS2	2
GO:0040013	negative regulation of locomotion	397/18866	0.008648657	0.02352744	PPARG/STAT3	2
GO:0051271	negative regulation of cellular component movement	400/18866	0.008775341	0.02360797	PPARG/STAT3	2
GO:0009150	purine ribonucleotide metabolic process	401/18866	0.008817759	0.02360797	HIF1A/STAT3	2
GO:0014850	response to muscle activity	24/18866	0.008872401	0.02360797	HIF1A	1
GO:0034695	response to prostaglandin E	24/18866	0.008872401	0.02360797	PPARG	1
GO:0036003	positive regulation of transcription from RNA polymerase II promoter in response to stress	24/18866	0.008872401	0.02360797	HIF1A	1
GO:0044321	response to leptin	24/18866	0.008872401	0.02360797	STAT3	1
GO:0045821	positive regulation of glycolytic process	24/18866	0.008872401	0.02360797	HIF1A	1
GO:0060396	growth hormone receptor signaling pathway	24/18866	0.008872401	0.02360797	STAT3	1
GO:0090335	regulation of brown fat cell differentiation	24/18866	0.008872401	0.02360797	PTGS2	1

GO:2000637	positive regulation of gene silencing by miRNA	24/18866	0.008872401	0.02360797	STAT3	1
GO:0043547	positive regulation of GTPase activity	406/18866	0.009031263	0.02395861	GSK3B/JUN	2
GO:0003309	type B pancreatic cell differentiation	25/18866	0.009240616	0.02395861	GSK3B	1
GO:0046697	decidualization	25/18866	0.009240616	0.02395861	PTGS2	1
GO:0060148	positive regulation of posttranscriptional gene silencing	25/18866	0.009240616	0.02395861	STAT3	1
GO:0060259	regulation of feeding behavior	25/18866	0.009240616	0.02395861	STAT3	1
GO:0060330	regulation of response to interferon-gamma	25/18866	0.009240616	0.02395861	PPARG	1
GO:0060334	regulation of interferon-gamma-mediated signaling pathway	25/18866	0.009240616	0.02395861	PPARG	1
GO:0060571	morphogenesis of an epithelial fold	25/18866	0.009240616	0.02395861	HIF1A	1
GO:0060575	intestinal epithelial cell differentiation	25/18866	0.009240616	0.02395861	HIF1A	1
GO:0070920	regulation of production of small RNA involved in gene silencing by RNA	25/18866	0.009240616	0.02395861	STAT3	1
GO:0071378	cellular response to growth hormone stimulus	25/18866	0.009240616	0.02395861	STAT3	1
GO:1905564	positive regulation of vascular endothelial cell proliferation	25/18866	0.009240616	0.02395861	STAT3	1
GO:0001503	ossification	412/18866	0.009290579	0.0240369	HIF1A/PTGS2	2
GO:0009259	ribonucleotide metabolic process	416/18866	0.009465335	0.02443704	HIF1A/STAT3	2
GO:0002068	glandular epithelial cell development	26/18866	0.009608713	0.02454663	GSK3B	1

GO:0030810	positive regulation of nucleotide biosynthetic process	26/18866	0.009608713	0.02454663	STAT3	1
GO:0035902	response to immobilization stress	26/18866	0.009608713	0.02454663	PPARG	1
GO:0051654	establishment of mitochondrion localization	26/18866	0.009608713	0.02454663	HIF1A	1
GO:1900373	positive regulation of purine nucleotide biosynthetic process	26/18866	0.009608713	0.02454663	STAT3	1
GO:0001558	regulation of cell growth	420/18866	0.00964159	0.02457898	GSK3B/PPARG	2
GO:0019693	ribose phosphate metabolic process	427/18866	0.009953634	0.0251173	HIF1A/STAT3	2
GO:0001963	synaptic transmission, dopaminergic	27/18866	0.009976692	0.0251173	PTGS2	1
GO:0032897	negative regulation of viral transcription	27/18866	0.009976692	0.0251173	JUN	1
GO:0072539	T-helper 17 cell differentiation	27/18866	0.009976692	0.0251173	STAT3	1
GO:1902175	regulation of oxidative stress-induced intrinsic apoptotic signaling pathway	27/18866	0.009976692	0.0251173	HIF1A	1
GO:1903203	regulation of oxidative stress-induced neuron death	27/18866	0.009976692	0.0251173	HIF1A	1
GO:0019216	regulation of lipid metabolic process	431/18866	0.010133994	0.02546061	PPARG/PTGS2	2
GO:0002360	T cell lineage commitment	28/18866	0.010344555	0.02567139	STAT3	1
GO:0035883	enteroendocrine cell differentiation	28/18866	0.010344555	0.02567139	GSK3B	1
GO:0045932	negative regulation of muscle contraction	28/18866	0.010344555	0.02567139	PTGS2	1
GO:1903579	negative regulation of ATP metabolic process	28/18866	0.010344555	0.02567139	STAT3	1

GO:1905563	negative regulation of vascular endothelial cell proliferation	28/18866	0.010344555	0.02567139	PPARG	1
GO:1990776	response to angiotensin	28/18866	0.010344555	0.02567139	PTGS2	1
GO:0006163	purine nucleotide metabolic process	442/18866	0.010637638	0.02626241	HIF1A/STAT3	2
GO:0042391	regulation of membrane potential	443/18866	0.010683979	0.02626241	GSK3B/JUN	2
GO:0048732	gland development	443/18866	0.010683979	0.02626241	HIF1A/JUN	2
GO:0001782	B cell homeostasis	29/18866	0.0107123	0.02626241	HIF1A	1
GO:0009435	NAD biosynthetic process	29/18866	0.0107123	0.02626241	PTGS2	1
GO:0036296	response to increased oxygen levels	29/18866	0.0107123	0.02626241	PPARG	1
GO:0009314	response to radiation	447/18866	0.010870264	0.02657435	HIF1A/PTGS2	2
GO:0001516	prostaglandin biosynthetic process	30/18866	0.011079929	0.02657435	PTGS2	1
GO:0005979	regulation of glycogen biosynthetic process	30/18866	0.011079929	0.02657435	GSK3B	1
GO:0010962	regulation of glucan biosynthetic process	30/18866	0.011079929	0.02657435	GSK3B	1
GO:0036475	neuron death in response to oxidative stress	30/18866	0.011079929	0.02657435	HIF1A	1
GO:0038111	interleukin-7-mediated signaling pathway	30/18866	0.011079929	0.02657435	STAT3	1
GO:0043457	regulation of cellular respiration	30/18866	0.011079929	0.02657435	HIF1A	1
GO:0046457	prostanoid biosynthetic process	30/18866	0.011079929	0.02657435	PTGS2	1
GO:0048147	negative regulation of fibroblast proliferation	30/18866	0.011079929	0.02657435	PPARG	1
GO:0070168	negative regulation of biomineral tissue development	30/18866	0.011079929	0.02657435	HIF1A	1

GO:0110150	negative regulation of biomineralization	30/18866	0.011079929	0.02657435	HIF1A	1
GO:0009896	positive regulation of catabolic process	454/18866	0.011199802	0.02680898	GSK3B/HIF1A	2
GO:0002675	positive regulation of acute inflammatory response	31/18866	0.01144744	0.0270293	PTGS2	1
GO:0032770	positive regulation of monooxygenase activity	31/18866	0.01144744	0.0270293	HIF1A	1
GO:0045987	positive regulation of smooth muscle contraction	31/18866	0.01144744	0.0270293	PTGS2	1
GO:0070229	negative regulation of lymphocyte apoptotic process	31/18866	0.01144744	0.0270293	HIF1A	1
GO:0071539	protein localization to centrosome	31/18866	0.01144744	0.0270293	GSK3B	1
GO:0072538	T-helper 17 type immune response	31/18866	0.01144744	0.0270293	STAT3	1
GO:1900181	negative regulation of protein localization to nucleus	31/18866	0.01144744	0.0270293	GSK3B	1
GO:0010743	regulation of macrophage derived foam cell differentiation	32/18866	0.011814834	0.02741763	PPARG	1
GO:0019359	nicotinamide nucleotide biosynthetic process	32/18866	0.011814834	0.02741763	PTGS2	1
GO:0019363	pyridine nucleotide biosynthetic process	32/18866	0.011814834	0.02741763	PTGS2	1
GO:0032350	regulation of hormone metabolic process	32/18866	0.011814834	0.02741763	HIF1A	1
GO:0034694	response to prostaglandin	32/18866	0.011814834	0.02741763	PPARG	1
GO:0046320	regulation of fatty acid oxidation	32/18866	0.011814834	0.02741763	PPARG	1
GO:0050849	negative regulation of calcium-mediated signaling	32/18866	0.011814834	0.02741763	GSK3B	1

GO:0051968	positive regulation of synaptic transmission, glutamatergic	32/18866	0.011814834	0.02741763	PTGS2	1
GO:0071295	cellular response to vitamin	32/18866	0.011814834	0.02741763	PPARG	1
GO:0060249	anatomical structure homeostasis	469/18866	0.011921054	0.02761143	HIF1A/PTGS2	2
GO:0072521	purine-containing compound metabolic process	472/18866	0.012067762	0.02784483	HIF1A/STAT3	2
GO:0051346	negative regulation of hydrolase activity	473/18866	0.012116847	0.02784483	GSK3B/PTGS2	2
GO:0010039	response to iron ion	33/18866	0.012182111	0.02784483	HIF1A	1
GO:0042755	eating behavior	33/18866	0.012182111	0.02784483	STAT3	1
GO:0048384	retinoic acid receptor signaling pathway	33/18866	0.012182111	0.02784483	PPARG	1
GO:1905048	regulation of metallopeptidase activity	33/18866	0.012182111	0.02784483	STAT3	1
GO:1905508	protein localization to microtubule organizing center	33/18866	0.012182111	0.02784483	GSK3B	1
GO:0043087	regulation of GTPase activity	481/18866	0.012512779	0.02815482	GSK3B/JUN	2
GO:0033198	response to ATP	34/18866	0.012549271	0.02815482	PTGS2	1
GO:0035633	maintenance of blood-brain barrier	34/18866	0.012549271	0.02815482	PTGS2	1
GO:0035774	positive regulation of insulin secretion involved in cellular response to glucose stimulus	34/18866	0.012549271	0.02815482	HIF1A	1
GO:0042759	long-chain fatty acid biosynthetic process	34/18866	0.012549271	0.02815482	PTGS2	1
GO:0045907	positive regulation of vasoconstriction	34/18866	0.012549271	0.02815482	PTGS2	1
GO:0055094	response to lipoprotein particle	34/18866	0.012549271	0.02815482	PPARG	1
GO:0070102	interleukin-6-mediated signaling pathway	34/18866	0.012549271	0.02815482	STAT3	1

GO:0070232	regulation of T cell apoptotic process	34/18866	0.012549271	0.02815482	HIF1A	1
GO:1902692	regulation of neuroblast proliferation	34/18866	0.012549271	0.02815482	HIF1A	1
GO:0042326	negative regulation of phosphorylation	484/18866	0.012662743	0.02835708	JUN/STAT3	2
GO:0050769	positive regulation of neurogenesis	485/18866	0.012712911	0.02840188	HIF1A/PPARG	2
GO:0019362	pyridine nucleotide metabolic process	35/18866	0.012916314	0.02840188	PTGS2	1
GO:0030947	regulation of vascular endothelial growth factor receptor signaling pathway	35/18866	0.012916314	0.02840188	HIF1A	1
GO:0034390	smooth muscle cell apoptotic process	35/18866	0.012916314	0.02840188	PPARG	1
GO:0034391	regulation of smooth muscle cell apoptotic process	35/18866	0.012916314	0.02840188	PPARG	1
GO:0046496	nicotinamide nucleotide metabolic process	35/18866	0.012916314	0.02840188	PTGS2	1
GO:0070884	regulation of calcineurin-NFAT signaling cascade	35/18866	0.012916314	0.02840188	GSK3B	1
GO:0072525	pyridine-containing compound biosynthetic process	35/18866	0.012916314	0.02840188	PTGS2	1
GO:0097009	energy homeostasis	35/18866	0.012916314	0.02840188	STAT3	1
GO:1901030	positive regulation of mitochondrial outer membrane permeabilization involved in apoptotic signaling pathway	35/18866	0.012916314	0.02840188	GSK3B	1
GO:0016049	cell growth	490/18866	0.012965101	0.0284577	GSK3B/PPARG	2
GO:0001893	maternal placenta development	36/18866	0.01328324	0.02879219	PTGS2	1
GO:0033280	response to vitamin D	36/18866	0.01328324	0.02879219	PTGS2	1
GO:0034405	response to fluid shear stress	36/18866	0.01328324	0.02879219	PTGS2	1
GO:0043368	positive T cell selection	36/18866	0.01328324	0.02879219	STAT3	1

GO:0070873	regulation of glycogen metabolic process	36/18866	0.01328324	0.02879219	GSK3B	1
GO:0071402	cellular response to lipoprotein particle stimulus	36/18866	0.01328324	0.02879219	PPARG	1
GO:0106056	regulation of calcineurin-mediated signaling	36/18866	0.01328324	0.02879219	GSK3B	1
GO:0090050	positive regulation of cell migration involved in sprouting angiogenesis	37/18866	0.013650049	0.02948217	PTGS2	1
GO:0098926	postsynaptic signal transduction	37/18866	0.013650049	0.02948217	STAT3	1
GO:0010661	positive regulation of muscle cell apoptotic process	38/18866	0.014016742	0.02990238	PPARG	1
GO:0010742	macrophage derived foam cell differentiation	38/18866	0.014016742	0.02990238	PPARG	1
GO:0032733	positive regulation of interleukin-10 production	38/18866	0.014016742	0.02990238	STAT3	1
GO:0032885	regulation of polysaccharide biosynthetic process	38/18866	0.014016742	0.02990238	GSK3B	1
GO:0071276	cellular response to cadmium ion	38/18866	0.014016742	0.02990238	JUN	1
GO:0090077	foam cell differentiation	38/18866	0.014016742	0.02990238	PPARG	1
GO:2000144	positive regulation of DNA-templated transcription, initiation	38/18866	0.014016742	0.02990238	JUN	1
GO:0060416	response to growth hormone	39/18866	0.014383317	0.03052376	STAT3	1
GO:1904706	negative regulation of vascular associated smooth muscle cell proliferation	39/18866	0.014383317	0.03052376	PPARG	1
GO:2000279	negative regulation of DNA biosynthetic process	39/18866	0.014383317	0.03052376	PPARG	1
GO:0042307	positive regulation of protein import into nucleus	40/18866	0.014749776	0.03108445	PTGS2	1

GO:0045740	positive regulation of DNA replication	40/18866	0.014749776	0.03108445	JUN	1
GO:0048713	regulation of oligodendrocyte differentiation	40/18866	0.014749776	0.03108445	PPARG	1
GO:1903427	negative regulation of reactive oxygen species biosynthetic process	40/18866	0.014749776	0.03108445	STAT3	1
GO:0007212	dopamine receptor signaling pathway	41/18866	0.015116118	0.03158282	GSK3B	1
GO:0046825	regulation of protein export from nucleus	41/18866	0.015116118	0.03158282	GSK3B	1
GO:0050434	positive regulation of viral transcription	41/18866	0.015116118	0.03158282	JUN	1
GO:0072524	pyridine-containing compound metabolic process	41/18866	0.015116118	0.03158282	PTGS2	1
GO:1902042	negative regulation of extrinsic apoptotic signaling pathway via death domain receptors	41/18866	0.015116118	0.03158282	GSK3B	1
GO:0071470	cellular response to osmotic stress	42/18866	0.015482343	0.03207245	PTGS2	1
GO:0098760	response to interleukin-7	42/18866	0.015482343	0.03207245	STAT3	1
GO:0098761	cellular response to interleukin-7	42/18866	0.015482343	0.03207245	STAT3	1
GO:1900371	regulation of purine nucleotide biosynthetic process	42/18866	0.015482343	0.03207245	STAT3	1
GO:1904591	positive regulation of protein import	42/18866	0.015482343	0.03207245	PTGS2	1
GO:0008631	intrinsic apoptotic signaling pathway in response to oxidative stress	43/18866	0.015848451	0.03255358	HIF1A	1

GO:0030808	regulation of nucleotide biosynthetic process	43/18866	0.015848451	0.03255358	STAT3	1
GO:0150077	regulation of neuroinflammatory response	43/18866	0.015848451	0.03255358	PTGS2	1
GO:1903146	regulation of autophagy of mitochondrion	43/18866	0.015848451	0.03255358	HIF1A	1
GO:2000648	positive regulation of stem cell proliferation	43/18866	0.015848451	0.03255358	HIF1A	1
GO:0031670	cellular response to nutrient	44/18866	0.016214443	0.0330264	PPARG	1
GO:0032881	regulation of polysaccharide metabolic process	44/18866	0.016214443	0.0330264	GSK3B	1
GO:0032965	regulation of collagen biosynthetic process	44/18866	0.016214443	0.0330264	PPARG	1
GO:0033173	calcineurin-NFAT signaling cascade	44/18866	0.016214443	0.0330264	GSK3B	1
GO:0045687	positive regulation of glial cell differentiation	44/18866	0.016214443	0.0330264	PPARG	1
GO:0031018	endocrine pancreas development	45/18866	0.016580318	0.0334356	GSK3B	1
GO:0032722	positive regulation of chemokine production	45/18866	0.016580318	0.0334356	HIF1A	1
GO:0045429	positive regulation of nitric oxide biosynthetic process	45/18866	0.016580318	0.0334356	PTGS2	1
GO:1901028	regulation of mitochondrial outer membrane permeabilization involved in apoptotic signaling pathway	45/18866	0.016580318	0.0334356	GSK3B	1
GO:1904646	cellular response to amyloid-beta	45/18866	0.016580318	0.0334356	GSK3B	1
GO:2001222	regulation of neuron migration	45/18866	0.016580318	0.0334356	STAT3	1
GO:0001754	eye photoreceptor cell differentiation	46/18866	0.016946076	0.03378103	STAT3	1
GO:0005978	glycogen biosynthetic process	46/18866	0.016946076	0.03378103	GSK3B	1
GO:0009250	glucan biosynthetic process	46/18866	0.016946076	0.03378103	GSK3B	1

GO:0032007	negative regulation of TOR signaling	46/18866	0.016946076	0.03378103	HIF1A	1
GO:0051646	mitochondrion localization	46/18866	0.016946076	0.03378103	HIF1A	1
GO:1904407	positive regulation of nitric oxide metabolic process	46/18866	0.016946076	0.03378103	PTGS2	1
GO:2000142	regulation of DNA-templated transcription, initiation	46/18866	0.016946076	0.03378103	JUN	1
GO:0008542	visual learning	47/18866	0.017311718	0.03439714	HIF1A	1
GO:0045933	positive regulation of muscle contraction	47/18866	0.017311718	0.03439714	PTGS2	1
GO:0009409	response to cold	48/18866	0.017677243	0.03489534	PPARG	1
GO:0031952	regulation of protein autophosphorylation	48/18866	0.017677243	0.03489534	JUN	1
GO:0051972	regulation of telomerase activity	48/18866	0.017677243	0.03489534	PPARG	1
GO:1900271	regulation of long-term synaptic potentiation	48/18866	0.017677243	0.03489534	GSK3B	1
GO:0002067	glandular epithelial cell differentiation	49/18866	0.018042652	0.03504771	GSK3B	1
GO:0006692	prostanoid metabolic process	49/18866	0.018042652	0.03504771	PTGS2	1
GO:0006693	prostaglandin metabolic process	49/18866	0.018042652	0.03504771	PTGS2	1
GO:0007595	lactation	49/18866	0.018042652	0.03504771	HIF1A	1
GO:0010712	regulation of collagen metabolic process	49/18866	0.018042652	0.03504771	PPARG	1
GO:0035196	production of miRNAs involved in gene silencing by miRNA	49/18866	0.018042652	0.03504771	STAT3	1
GO:0045058	T cell selection	49/18866	0.018042652	0.03504771	STAT3	1
GO:0070231	T cell apoptotic process	49/18866	0.018042652	0.03504771	HIF1A	1
GO:0097720	calcineurin-mediated signaling	49/18866	0.018042652	0.03504771	GSK3B	1
GO:2000107	negative regulation of leukocyte apoptotic process	49/18866	0.018042652	0.03504771	HIF1A	1
GO:0001774	microglial cell activation	50/18866	0.018407944	0.03547395	JUN	1

GO:0002269	leukocyte activation involved in inflammatory response	50/18866	0.018407944	0.03547395	JUN	1
GO:0048546	digestive tract morphogenesis	50/18866	0.018407944	0.03547395	HIF1A	1
GO:0050873	brown fat cell differentiation	50/18866	0.018407944	0.03547395	PTGS2	1
GO:1904707	positive regulation of vascular associated smooth muscle cell proliferation	50/18866	0.018407944	0.03547395	JUN	1
GO:0006984	ER-nucleus signaling pathway	51/18866	0.018773119	0.03600649	GSK3B	1
GO:0048146	positive regulation of fibroblast proliferation	51/18866	0.018773119	0.03600649	JUN	1
GO:0071354	cellular response to interleukin-6	51/18866	0.018773119	0.03600649	STAT3	1
GO:0010883	regulation of lipid storage	52/18866	0.019138178	0.03653379	PPARG	1
GO:0031103	axon regeneration	52/18866	0.019138178	0.03653379	JUN	1
GO:0050999	regulation of nitric-oxide synthase activity	52/18866	0.019138178	0.03653379	HIF1A	1
GO:0007632	visual behavior	53/18866	0.019503121	0.03699812	HIF1A	1
GO:0032964	collagen biosynthetic process	53/18866	0.019503121	0.03699812	PPARG	1
GO:0043392	negative regulation of DNA binding	53/18866	0.019503121	0.03699812	JUN	1
GO:0072698	protein localization to microtubule cytoskeleton	53/18866	0.019503121	0.03699812	GSK3B	1
GO:0006754	ATP biosynthetic process	54/18866	0.019867948	0.03734069	STAT3	1
GO:0030857	negative regulation of epithelial cell differentiation	54/18866	0.019867948	0.03734069	GSK3B	1
GO:0031050	dsRNA processing	54/18866	0.019867948	0.03734069	STAT3	1
GO:0032757	positive regulation of interleukin-8 production	54/18866	0.019867948	0.03734069	STAT3	1
GO:0070918	production of small RNA involved in gene silencing by RNA	54/18866	0.019867948	0.03734069	STAT3	1

GO:2000179	positive regulation of neural precursor cell proliferation	54/18866	0.019867948	0.03734069	HIF1A	1
GO:0001954	positive regulation of cell-matrix adhesion	55/18866	0.020232657	0.03773453	GSK3B	1
GO:0042304	regulation of fatty acid biosynthetic process	55/18866	0.020232657	0.03773453	PTGS2	1
GO:0051353	positive regulation of oxidoreductase activity	55/18866	0.020232657	0.03773453	HIF1A	1
GO:0070741	response to interleukin-6	55/18866	0.020232657	0.03773453	STAT3	1
GO:0097345	mitochondrial outer membrane permeabilization	55/18866	0.020232657	0.03773453	GSK3B	1
GO:0001755	neural crest cell migration	56/18866	0.020597251	0.03823856	HIF1A	1
GO:0070228	regulation of lymphocyte apoptotic process	56/18866	0.020597251	0.03823856	HIF1A	1
GO:1904645	response to amyloid-beta	56/18866	0.020597251	0.03823856	GSK3B	1
GO:0042743	hydrogen peroxide metabolic process	57/18866	0.020961729	0.03856197	STAT3	1
GO:0044380	protein localization to cytoskeleton	57/18866	0.020961729	0.03856197	GSK3B	1
GO:0045747	positive regulation of Notch signaling pathway	57/18866	0.020961729	0.03856197	STAT3	1
GO:0048016	inositol phosphate-mediated signaling	57/18866	0.020961729	0.03856197	GSK3B	1
GO:0061178	regulation of insulin secretion involved in cellular response to glucose stimulus	57/18866	0.020961729	0.03856197	HIF1A	1
GO:0071398	cellular response to fatty acid	57/18866	0.020961729	0.03856197	PPARG	1
GO:0002763	positive regulation of myeloid leukocyte differentiation	58/18866	0.02132609	0.03893773	JUN	1
GO:0007566	embryo implantation	58/18866	0.02132609	0.03893773	PTGS2	1
GO:0019369	arachidonic acid metabolic process	58/18866	0.02132609	0.03893773	PTGS2	1

GO:1900408	negative regulation of cellular response to oxidative stress	58/18866	0.02132609	0.03893773	HIF1A	1
GO:1903202	negative regulation of oxidative stress-induced cell death	58/18866	0.02132609	0.03893773	HIF1A	1
GO:0006636	unsaturated fatty acid biosynthetic process	59/18866	0.021690334	0.03924918	PTGS2	1
GO:0031102	neuron projection regeneration	59/18866	0.021690334	0.03924918	JUN	1
GO:0042306	regulation of protein import into nucleus	59/18866	0.021690334	0.03924918	PTGS2	1
GO:0043525	positive regulation of neuron apoptotic process	59/18866	0.021690334	0.03924918	JUN	1
GO:0046456	icosanoid biosynthetic process	59/18866	0.021690334	0.03924918	PTGS2	1
GO:0098930	axonal transport	59/18866	0.021690334	0.03924918	HIF1A	1
GO:0001836	release of cytochrome c from mitochondria	60/18866	0.022054463	0.03938066	JUN	1
GO:0019229	regulation of vasoconstriction	60/18866	0.022054463	0.03938066	PTGS2	1
GO:0032515	negative regulation of phosphoprotein phosphatase activity	60/18866	0.022054463	0.03938066	GSK3B	1
GO:0032653	regulation of interleukin-10 production	60/18866	0.022054463	0.03938066	STAT3	1
GO:0042093	T-helper cell differentiation	60/18866	0.022054463	0.03938066	STAT3	1
GO:0043388	positive regulation of DNA binding	60/18866	0.022054463	0.03938066	PPARG	1
GO:0061900	glial cell activation	60/18866	0.022054463	0.03938066	JUN	1
GO:1902883	negative regulation of response to oxidative stress	60/18866	0.022054463	0.03938066	HIF1A	1
GO:1903428	positive regulation of reactive oxygen species biosynthetic process	60/18866	0.022054463	0.03938066	PTGS2	1

GO:1902041	regulation of extrinsic apoptotic signaling pathway via death domain receptors	61/18866	0.022418476	0.03991342	GSK3B	1
GO:1902110	positive regulation of mitochondrial membrane permeability involved in apoptotic process	61/18866	0.022418476	0.03991342	GSK3B	1
GO:0002260	lymphocyte homeostasis	62/18866	0.022782372	0.0401498	HIF1A	1
GO:0002294	CD4-positive, alpha-beta T cell differentiation involved in immune response	62/18866	0.022782372	0.0401498	STAT3	1
GO:0007405	neuroblast proliferation	62/18866	0.022782372	0.0401498	HIF1A	1
GO:0010803	regulation of tumor necrosis factor-mediated signaling pathway	62/18866	0.022782372	0.0401498	CASP1	1
GO:0045824	negative regulation of innate immune response	62/18866	0.022782372	0.0401498	PPARG	1
GO:0051851	modulation by host of symbiont process	62/18866	0.022782372	0.0401498	JUN	1
GO:1904589	regulation of protein import	62/18866	0.022782372	0.0401498	PTGS2	1
GO:0002287	alpha-beta T cell activation involved in immune response	63/18866	0.023146152	0.04049744	STAT3	1
GO:0002293	alpha-beta T cell differentiation involved in immune response	63/18866	0.023146152	0.04049744	STAT3	1
GO:0032613	interleukin-10 production	63/18866	0.023146152	0.04049744	STAT3	1
GO:0046530	photoreceptor cell differentiation	63/18866	0.023146152	0.04049744	STAT3	1
GO:1902686	mitochondrial outer membrane permeabilization involved in programmed cell death	63/18866	0.023146152	0.04049744	GSK3B	1
GO:0001947	heart looping	64/18866	0.023509816	0.04089834	HIF1A	1
GO:0046686	response to cadmium ion	64/18866	0.023509816	0.04089834	JUN	1

GO:0051926	negative regulation of calcium ion transport	64/18866	0.023509816	0.04089834	PTGS2	1
GO:0060135	maternal process involved in female pregnancy	64/18866	0.023509816	0.04089834	PTGS2	1
GO:0006940	regulation of smooth muscle contraction	65/18866	0.023873364	0.041119	PTGS2	1
GO:0009206	purine ribonucleoside triphosphate biosynthetic process	65/18866	0.023873364	0.041119	STAT3	1
GO:0035308	negative regulation of protein dephosphorylation	65/18866	0.023873364	0.041119	GSK3B	1
GO:0035773	insulin secretion involved in cellular response to glucose stimulus	65/18866	0.023873364	0.041119	HIF1A	1
GO:0035794	positive regulation of mitochondrial membrane permeability	65/18866	0.023873364	0.041119	GSK3B	1
GO:0046782	regulation of viral transcription	65/18866	0.023873364	0.041119	JUN	1
GO:1905953	negative regulation of lipid localization	65/18866	0.023873364	0.041119	PPARG	1
GO:0009145	purine nucleoside triphosphate biosynthetic process	66/18866	0.024236797	0.04156833	STAT3	1
GO:0032768	regulation of monooxygenase activity	66/18866	0.024236797	0.04156833	HIF1A	1
GO:1902108	regulation of mitochondrial membrane permeability involved in apoptotic process	66/18866	0.024236797	0.04156833	GSK3B	1
GO:0071300	cellular response to retinoic acid	67/18866	0.024600113	0.04195475	PPARG	1
GO:1900015	regulation of cytokine production involved in inflammatory response	67/18866	0.024600113	0.04195475	STAT3	1

GO:1903672	positive regulation of sprouting angiogenesis	67/18866	0.024600113	0.04195475	PTGS2	1
GO:1905710	positive regulation of membrane permeability	67/18866	0.024600113	0.04195475	GSK3B	1
GO:0016239	positive regulation of macroautophagy	68/18866	0.024963313	0.0422189	HIF1A	1
GO:0032642	regulation of chemokine production	68/18866	0.024963313	0.0422189	HIF1A	1
GO:0040014	regulation of multicellular organism growth	68/18866	0.024963313	0.0422189	STAT3	1
GO:0042446	hormone biosynthetic process	68/18866	0.024963313	0.0422189	HIF1A	1
GO:0045428	regulation of nitric oxide biosynthetic process	68/18866	0.024963313	0.0422189	PTGS2	1
GO:0050766	positive regulation of phagocytosis	68/18866	0.024963313	0.0422189	PPARG	1
GO:0048662	negative regulation of smooth muscle cell proliferation	69/18866	0.025326397	0.04271415	PPARG	1
GO:0061371	determination of heart left/right asymmetry	69/18866	0.025326397	0.04271415	HIF1A	1
GO:0002534	cytokine production involved in inflammatory response	70/18866	0.025689365	0.04302792	STAT3	1
GO:0003143	embryonic heart tube morphogenesis	70/18866	0.025689365	0.04302792	HIF1A	1
GO:0006879	cellular iron ion homeostasis	70/18866	0.025689365	0.04302792	HIF1A	1
GO:0008088	axo-dendritic transport	70/18866	0.025689365	0.04302792	HIF1A	1
GO:0050795	regulation of behavior	70/18866	0.025689365	0.04302792	STAT3	1
GO:0002292	T cell differentiation involved in immune response	71/18866	0.026052218	0.04321896	STAT3	1
GO:0009201	ribonucleoside triphosphate biosynthetic process	71/18866	0.026052218	0.04321896	STAT3	1
GO:0014823	response to activity	71/18866	0.026052218	0.04321896	HIF1A	1
GO:0032371	regulation of sterol transport	71/18866	0.026052218	0.04321896	PPARG	1

GO:0033692	cellular polysaccharide biosynthetic process	71/18866	0.026052218	0.04321896	GSK3B	1
GO:0042531	positive regulation of tyrosine phosphorylation of STAT protein	71/18866	0.026052218	0.04321896	STAT3	1
GO:0061180	mammary gland epithelium development	71/18866	0.026052218	0.04321896	HIF1A	1
GO:0003208	cardiac ventricle morphogenesis	73/18866	0.026777575	0.04418118	HIF1A	1
GO:0005977	glycogen metabolic process	73/18866	0.026777575	0.04418118	GSK3B	1
GO:0051966	regulation of synaptic transmission, glutamatergic	73/18866	0.026777575	0.04418118	PTGS2	1
GO:0070227	lymphocyte apoptotic process	73/18866	0.026777575	0.04418118	HIF1A	1
GO:0006073	cellular glucan metabolic process	74/18866	0.02714008	0.04459067	GSK3B	1
GO:0032024	positive regulation of insulin secretion	74/18866	0.02714008	0.04459067	HIF1A	1
GO:0044042	glucan metabolic process	74/18866	0.02714008	0.04459067	GSK3B	1
GO:0015909	long-chain fatty acid transport	75/18866	0.027502469	0.04459067	PPARG	1
GO:0031100	animal organ regeneration	75/18866	0.027502469	0.04459067	PPARG	1
GO:0032602	chemokine production	75/18866	0.027502469	0.04459067	HIF1A	1
GO:0035019	somatic stem cell population maintenance	75/18866	0.027502469	0.04459067	STAT3	1
GO:0038034	signal transduction in absence of ligand	75/18866	0.027502469	0.04459067	GSK3B	1
GO:0043627	response to estrogen	75/18866	0.027502469	0.04459067	PPARG	1
GO:0045685	regulation of glial cell differentiation	75/18866	0.027502469	0.04459067	PPARG	1
GO:0050805	negative regulation of synaptic transmission	75/18866	0.027502469	0.04459067	PTGS2	1
GO:0097192	extrinsic apoptotic signaling pathway in absence of ligand	75/18866	0.027502469	0.04459067	GSK3B	1

GO:1901983	regulation of protein acetylation	75/18866	0.027502469	0.04459067	GSK3B	1
GO:0008306	associative learning	76/18866	0.027864743	0.04499804	HIF1A	1
GO:0072091	regulation of stem cell proliferation	76/18866	0.027864743	0.04499804	HIF1A	1
GO:1903524	positive regulation of blood circulation	76/18866	0.027864743	0.04499804	PTGS2	1
GO:0014015	positive regulation of gliogenesis	77/18866	0.028226901	0.04534202	PPARG	1
GO:0030500	regulation of bone mineralization	77/18866	0.028226901	0.04534202	HIF1A	1
GO:0061418	regulation of transcription from RNA polymerase II promoter in response to hypoxia	77/18866	0.028226901	0.04534202	HIF1A	1
GO:1903201	regulation of oxidative stress-induced cell death	77/18866	0.028226901	0.04534202	HIF1A	1
GO:0000271	polysaccharide biosynthetic process	78/18866	0.028588943	0.0456822	GSK3B	1
GO:0042310	vasoconstriction	78/18866	0.028588943	0.0456822	PTGS2	1
GO:0043367	CD4-positive, alpha-beta T cell differentiation	78/18866	0.028588943	0.0456822	STAT3	1
GO:0060395	SMAD protein signal transduction	78/18866	0.028588943	0.0456822	JUN	1
GO:0000422	autophagy of mitochondrion	79/18866	0.02895087	0.04589864	HIF1A	1
GO:0006809	nitric oxide biosynthetic process	79/18866	0.02895087	0.04589864	PTGS2	1
GO:0009205	purine ribonucleoside triphosphate metabolic process	79/18866	0.02895087	0.04589864	STAT3	1
GO:0031016	pancreas development	79/18866	0.02895087	0.04589864	GSK3B	1
GO:0061726	mitochondrion disassembly	79/18866	0.02895087	0.04589864	HIF1A	1
GO:1900034	regulation of cellular response to heat	79/18866	0.02895087	0.04589864	GSK3B	1

GO:0014032	neural crest cell development	80/18866	0.029312681	0.04617127	HIF1A	1
GO:0035050	embryonic heart tube development	80/18866	0.029312681	0.04617127	HIF1A	1
GO:0048678	response to axon injury	80/18866	0.029312681	0.04617127	JUN	1
GO:1900182	positive regulation of protein localization to nucleus	80/18866	0.029312681	0.04617127	PTGS2	1
GO:2000300	regulation of synaptic vesicle exocytosis	80/18866	0.029312681	0.04617127	GSK3B	1
GO:0001937	negative regulation of endothelial cell proliferation	81/18866	0.029674376	0.04644021	PPARG	1
GO:0001960	negative regulation of cytokine-mediated signaling pathway	81/18866	0.029674376	0.04644021	PPARG	1
GO:0019915	lipid storage	81/18866	0.029674376	0.04644021	PPARG	1
GO:0021766	hippocampus development	81/18866	0.029674376	0.04644021	GSK3B	1
GO:0034644	cellular response to UV	81/18866	0.029674376	0.04644021	PTGS2	1
GO:0009142	nucleoside triphosphate biosynthetic process	82/18866	0.030035956	0.04694566	STAT3	1
GO:0014855	striated muscle cell proliferation	83/18866	0.030397421	0.04732812	STAT3	1
GO:0048708	astrocyte differentiation	83/18866	0.030397421	0.04732812	STAT3	1
GO:0071277	cellular response to calcium ion	83/18866	0.030397421	0.04732812	JUN	1
GO:0014031	mesenchymal cell development	84/18866	0.03075877	0.04758609	HIF1A	1
GO:0030279	negative regulation of ossification	84/18866	0.03075877	0.04758609	HIF1A	1
GO:0045913	positive regulation of carbohydrate metabolic process	84/18866	0.03075877	0.04758609	HIF1A	1
GO:0046209	nitric oxide metabolic process	84/18866	0.03075877	0.04758609	PTGS2	1
GO:0048864	stem cell development	84/18866	0.03075877	0.04758609	HIF1A	1
GO:0006970	response to osmotic stress	85/18866	0.031120003	0.04778021	PTGS2	1
GO:0009199	ribonucleoside triphosphate metabolic process	85/18866	0.031120003	0.04778021	STAT3	1

GO:0010507	negative regulation of autophagy	85/18866	0.031120003	0.04778021	STAT3	1
GO:0032092	positive regulation of protein binding	85/18866	0.031120003	0.04778021	GSK3B	1
GO:0043154	negative regulation of cysteine-type endopeptidase activity involved in apoptotic process	85/18866	0.031120003	0.04778021	PTGS2	1
GO:0072384	organelle transport along microtubule	85/18866	0.031120003	0.04778021	HIF1A	1
GO:0001776	leukocyte homeostasis	86/18866	0.031481121	0.04791119	HIF1A	1
GO:0006112	energy reserve metabolic process	86/18866	0.031481121	0.04791119	GSK3B	1
GO:0006919	activation of cysteine-type endopeptidase activity involved in apoptotic process	86/18866	0.031481121	0.04791119	PPARG	1
GO:0034637	cellular carbohydrate biosynthetic process	86/18866	0.031481121	0.04791119	GSK3B	1
GO:0042509	regulation of tyrosine phosphorylation of STAT protein	86/18866	0.031481121	0.04791119	STAT3	1
GO:0097756	negative regulation of blood vessel diameter	86/18866	0.031481121	0.04791119	PTGS2	1
GO:2000106	regulation of leukocyte apoptotic process	86/18866	0.031481121	0.04791119	HIF1A	1
GO:0009060	aerobic respiration	87/18866	0.031842124	0.0479802	HIF1A	1
GO:0009144	purine nucleoside triphosphate metabolic process	87/18866	0.031842124	0.0479802	STAT3	1
GO:0032436	positive regulation of proteasomal ubiquitin-dependent protein catabolic process	87/18866	0.031842124	0.0479802	GSK3B	1
GO:0055072	iron ion homeostasis	87/18866	0.031842124	0.0479802	HIF1A	1

GO:0060291	long-term synaptic potentiation	87/18866	0.031842124	0.0479802	GSK3B	1
GO:0060761	negative regulation of response to cytokine stimulus	87/18866	0.031842124	0.0479802	PPARG	1
GO:0090049	regulation of cell migration involved in sprouting angiogenesis	87/18866	0.031842124	0.0479802	PTGS2	1
GO:2001057	reactive nitrogen species metabolic process	87/18866	0.031842124	0.0479802	PTGS2	1
GO:0032755	positive regulation of interleukin-6 production	88/18866	0.032203012	0.04834427	STAT3	1
GO:0046889	positive regulation of lipid biosynthetic process	88/18866	0.032203012	0.04834427	PTGS2	1
GO:0070098	chemokine-mediated signaling pathway	88/18866	0.032203012	0.04834427	HIF1A	1
GO:0001892	embryonic placenta development	89/18866	0.032563784	0.04858596	HIF1A	1
GO:0007260	tyrosine phosphorylation of STAT protein	89/18866	0.032563784	0.04858596	STAT3	1
GO:0008625	extrinsic apoptotic signaling pathway via death domain receptors	89/18866	0.032563784	0.04858596	GSK3B	1
GO:0014033	neural crest cell differentiation	89/18866	0.032563784	0.04858596	HIF1A	1
GO:0043537	negative regulation of blood vessel endothelial cell migration	89/18866	0.032563784	0.04858596	PPARG	1
GO:0046427	positive regulation of receptor signaling pathway via JAK-STAT	90/18866	0.032924441	0.04900382	STAT3	1
GO:1903351	cellular response to dopamine	90/18866	0.032924441	0.04900382	GSK3B	1
GO:0051899	membrane depolarization	91/18866	0.033284982	0.0492391	JUN	1
GO:0060333	interferon-gamma-mediated signaling pathway	91/18866	0.033284982	0.0492391	PPARG	1

GO:0097306	cellular response to alcohol	91/18866	0.033284982	0.0492391	PPARG	1
GO:1903350	response to dopamine	91/18866	0.033284982	0.0492391	GSK3B	1
GO:2000177	regulation of neural precursor cell proliferation	91/18866	0.033284982	0.0492391	HIF1A	1
GO:0106027	neuron projection organization	92/18866	0.033645408	0.04965148	GSK3B	1
GO:1900407	regulation of cellular response to oxidative stress	92/18866	0.033645408	0.04965148	HIF1A	1
GO:0002532	production of molecular mediator involved in inflammatory response	93/18866	0.03400572	0.04988053	STAT3	1
GO:0032677	regulation of interleukin-8 production	93/18866	0.03400572	0.04988053	STAT3	1
GO:0051702	interaction with symbiont	93/18866	0.03400572	0.04988053	JUN	1
GO:1904894	positive regulation of receptor signaling pathway via STAT	93/18866	0.03400572	0.04988053	STAT3	1
GO:2000117	negative regulation of cysteine-type endopeptidase activity	93/18866	0.03400572	0.04988053	PTGS2	1

Supplementary Table 4: GO Cell Components

ID	Description	BgRatio	pvalue	p.adjust	geneID	Count
GO:0090575	RNA polymerase II transcription regulator co	161/19559	1.52E-07	5.01E-06	HIF1A/JUN/PPARG/STAT3	4
GO:0005667	transcription regulator complex	413/19559	6.52E-06	0.0001076	HIF1A/JUN/PPARG/STAT3	4
GO:0030877	beta-catenin destruction complex	12/19559	0.004287458	0.03537153	GSK3B	1
GO:1990909	Wnt signalosome	12/19559	0.004287458	0.03537153	GSK3B	1
GO:0061702	inflammasome complex	17/19559	0.006069243	0.03690322	CASP1	1
GO:0098978	glutamatergic synapse	361/19559	0.006709676	0.03690322	GSK3B/STAT3	2
GO:0005640	nuclear outer membrane	28/19559	0.009979552	0.04704646	PTGS2	1
GO:0005719	nuclear euchromatin	34/19559	0.012106885	0.0499409	JUN	1

Supplementary Table 5: GO Molecular Functions

ID	Description	BgRatio	pvalue	p.adjust	geneID	Count
GO:0061629	RNA polymerase II-specific DNA-binding trans	267/18352	1.29E-08	1.12E-06	GSK3B/HIF1A/JUN/PPARG/STA	5
GO:0140297	DNA-binding transcription factor binding	347/18352	4.78E-08	2.08E-06	GSK3B/HIF1A/JUN/PPARG/STA	5
GO:0035257	nuclear hormone receptor binding	144/18352	1.62E-05	0.00046929	HIF1A/PPARG/STAT3	3
GO:0051427	hormone receptor binding	177/18352	3.00E-05	0.00065265	HIF1A/PPARG/STAT3	3
GO:0001103	RNA polymerase II repressing transcription f	35/18352	7.38E-05	0.00128342	PPARG/STAT3	2
GO:0031625	ubiquitin protein ligase binding	297/18352	0.000139953	0.00146283	GSK3B/HIF1A/JUN	3
GO:0070888	E-box binding	50/18352	0.000151445	0.00146283	HIF1A/PPARG	2
GO:0004879	nuclear receptor activity	52/18352	0.000163871	0.00146283	PPARG/STAT3	2
GO:0098531	ligand-activated transcription factor activi	52/18352	0.000163871	0.00146283	PPARG/STAT3	2
GO:0044389	ubiquitin-like protein ligase binding	316/18352	0.000168142	0.00146283	GSK3B/HIF1A/JUN	3
GO:0002039	p53 binding	66/18352	0.000264412	0.00209126	GSK3B/HIF1A	2
GO:0070491	repressing transcription factor binding	74/18352	0.000332465	0.00241037	PPARG/STAT3	2
GO:0033613	activating transcription factor binding	80/18352	0.000388539	0.00247517	JUN/PPARG	2
GO:0035258	steroid hormone receptor binding	81/18352	0.000398303	0.00247517	PPARG/STAT3	2
GO:0016922	nuclear receptor binding	101/18352	0.000618557	0.00358763	PPARG/STAT3	2
GO:0019903	protein phosphatase binding	149/18352	0.001338785	0.00727964	PPARG/STAT3	2
GO:0019902	phosphatase binding	194/18352	0.002254554	0.01153801	PPARG/STAT3	2
GO:0004955	prostaglandin receptor activity	10/18352	0.00380869	0.01743979	PPARG	1
GO:0097199	cysteine-type endopeptidase activity involve	10/18352	0.00380869	0.01743979	CASP1	1
GO:0004954	prostanoid receptor activity	11/18352	0.004188874	0.01795555	PPARG	1
GO:0034452	dynactin binding	12/18352	0.004568934	0.01795555	GSK3B	1
GO:0034236	protein kinase A catalytic subunit binding	13/18352	0.00494887	0.01795555	GSK3B	1
GO:0035497	cAMP response element binding	14/18352	0.005328681	0.01795555	JUN	1
GO:0004953	icosanoid receptor activity	15/18352	0.005708368	0.01795555	PPARG	1
GO:0035259	glucocorticoid receptor binding	15/18352	0.005708368	0.01795555	STAT3	1
GO:0036041	long-chain fatty acid binding	15/18352	0.005708368	0.01795555	PPARG	1
GO:0097153	cysteine-type endopeptidase activity involve	15/18352	0.005708368	0.01795555	CASP1	1
GO:0050700	CARD domain binding	16/18352	0.006087931	0.01795555	CASP1	1
GO:0071837	HMG box domain binding	16/18352	0.006087931	0.01795555	JUN	1

GO:0008656	cysteine-type endopeptidase activator activi	17/18352	0.006467369	0.01795555	CASP1	1
GO:0001227	DNA-binding transcription repressor activity	335/18352	0.006566229	0.01795555	JUN/PPARG	2
GO:0001217	DNA-binding transcription repressor activity	336/18352	0.006604341	0.01795555	JUN/PPARG	2
GO:0046965	retinoid X receptor binding	18/18352	0.006846684	0.01805035	PPARG	1
GO:0016505	peptidase activator activity involved in apo	20/18352	0.00760494	0.0194597	CASP1	1
GO:0050321	tau-protein kinase activity	22/18352	0.0083627	0.02078728	GSK3B	1
GO:0070412	R-SMAD binding	23/18352	0.008741394	0.02112504	JUN	1
GO:0016702	oxidoreductase activity, acting on single do	25/18352	0.00949841	0.02230447	PTGS2	1
GO:0016701	oxidoreductase activity, acting on single do	26/18352	0.009876732	0.02230447	PTGS2	1
GO:0042974	retinoic acid receptor binding	27/18352	0.010254931	0.02230447	PPARG	1
GO:0051393	alpha-actinin binding	27/18352	0.010254931	0.02230447	PPARG	1
GO:0035035	histone acetyltransferase binding	28/18352	0.010633005	0.02256272	HIF1A	1
GO:1901567	fatty acid derivative binding	29/18352	0.011010956	0.02280841	PPARG	1
GO:0051059	NF-kappaB binding	31/18352	0.011766486	0.02380661	GSK3B	1
GO:0042805	actinin binding	36/18352	0.013653147	0.026996	PPARG	1
GO:0005504	fatty acid binding	39/18352	0.014783662	0.02858175	PPARG	1
GO:0016504	peptidase activator activity	40/18352	0.015160253	0.02867265	CASP1	1
GO:0030331	estrogen receptor binding	42/18352	0.015913065	0.02884243	PPARG	1
GO:0051879	Hsp90 protein binding	42/18352	0.015913065	0.02884243	HIF1A	1
GO:0043028	cysteine-type endopeptidase regulator activi	43/18352	0.016289286	0.02892179	CASP1	1
GO:0048156	tau protein binding	45/18352	0.017041359	0.02965196	GSK3B	1
GO:0001102	RNA polymerase II activating transcription f	47/18352	0.017792938	0.02976895	JUN	1
GO:0048020	CCR chemokine receptor binding	47/18352	0.017792938	0.02976895	STAT3	1
GO:0051018	protein kinase A binding	51/18352	0.019294619	0.0316723	GSK3B	1
GO:0004601	peroxidase activity	52/18352	0.019669732	0.03169012	PTGS2	1
GO:0001085	RNA polymerase II transcription factor bindi	56/18352	0.021168953	0.03288211	GSK3B	1
GO:0016684	oxidoreductase activity, acting on peroxide	56/18352	0.021168953	0.03288211	PTGS2	1
GO:0043621	protein self-association	57/18352	0.021543452	0.03288211	PPARG	1
GO:0042379	chemokine receptor binding	70/18352	0.026400763	0.03960114	STAT3	1
GO:0033293	monocarboxylic acid binding	72/18352	0.027146204	0.04002915	PPARG	1
GO:0046332	SMAD binding	79/18352	0.029751396	0.04313952	JUN	1
GO:0008013	beta-catenin binding	85/18352	0.031979656	0.04539521	GSK3B	1
GO:0016209	antioxidant activity	86/18352	0.032350606	0.04539521	PTGS2	1

GO:0051213	dioxygenase activity	91/18352	0.034203529	0.04723344	PTGS2	1

Supplementary Table 6: KEGG pathway

ID	Description	BgRatio	pvalue	p.adjust	geneID	Count
hsa04657	IL-17 signaling pathway	94/8096	5.13E-05	0.00151334	2932/3725/5743	3
hsa04625	C-type lectin receptor signaling pathway	104/8096	6.94E-05	0.00152858	834/3725/5743	3
hsa04659	Th17 cell differentiation	108/8096	7.77E-05	0.00152858	3091/3725/6774	3
hsa04917	Prolactin signaling pathway	70/8096	0.001504866	0.01173667	2932/6774	2
hsa01521	EGFR tyrosine kinase inhibitor resistance	79/8096	0.001912747	0.01187917	2932/6774	2
hsa04662	B cell receptor signaling pathway	82/8096	0.00205919	0.01213546	2932/3725	2
hsa04012	ErbB signaling pathway	85/8096	0.00221084	0.01213546	2932/3725	2
hsa04660	T cell receptor signaling pathway	104/8096	0.003290925	0.01553316	2932/3725	2
hsa04931	Insulin resistance	108/8096	0.003544348	0.01577312	2932/6774	2
hsa04066	HIF-1 signaling pathway	109/8096	0.003609104	0.01577312	3091/6774	2
hsa04668	TNF signaling pathway	112/8096	0.003806721	0.01604261	3725/5743	2
hsa04722	Neurotrophin signaling pathway	119/8096	0.004287272	0.01686085	2932/3725	2
hsa04935	Growth hormone synthesis, secretion and acti	119/8096	0.004287272	0.01686085	2932/6774	2
hsa04919	Thyroid hormone signaling pathway	121/8096	0.004429546	0.01686085	2932/3091	2
hsa04550	Signaling pathways regulating pluripotency o	143/8096	0.006138478	0.02194971	2932/6774	2
hsa04921	Oxytocin signaling pathway	154/8096	0.007090372	0.02390468	3725/5743	2
hsa04310	Wnt signaling pathway	166/8096	0.008201411	0.02481452	2932/3725	2
hsa04621	NOD-like receptor signaling pathway	185/8096	0.010112613	0.02983221	834/3725	2
hsa04062	Chemokine signaling pathway	192/8096	0.010862926	0.03126403	2932/6774	2
hsa04510	Focal adhesion	201/8096	0.011863578	0.03160651	2932/3725	2

Related signaling pathway supplement

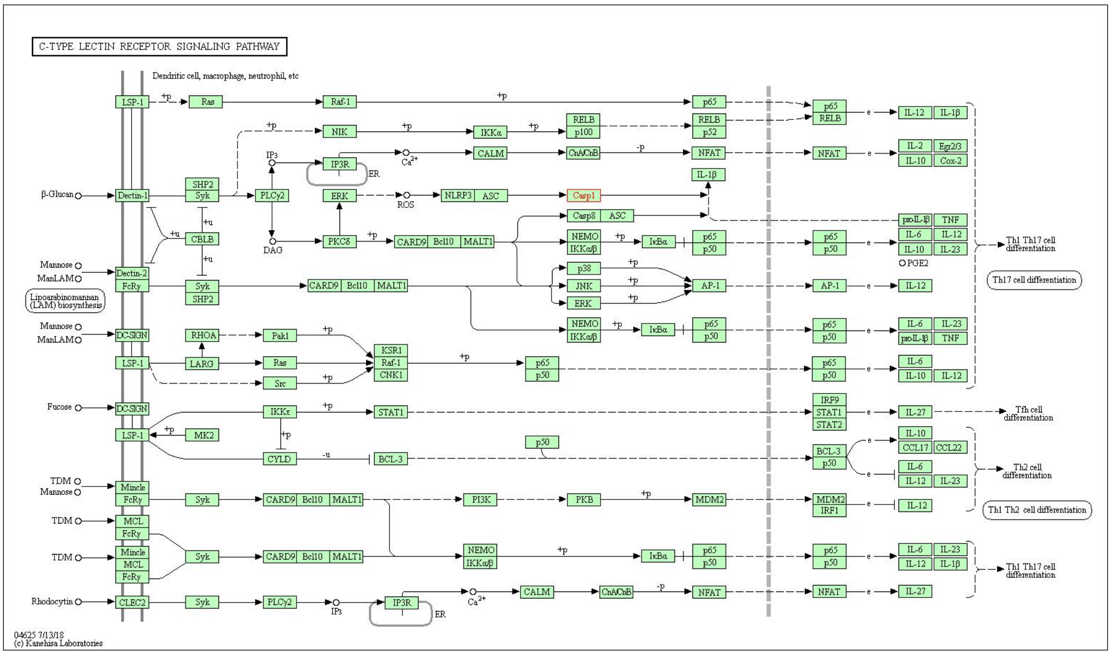

Supplementary Figure 1 Type C lectin receptor signaling pathway

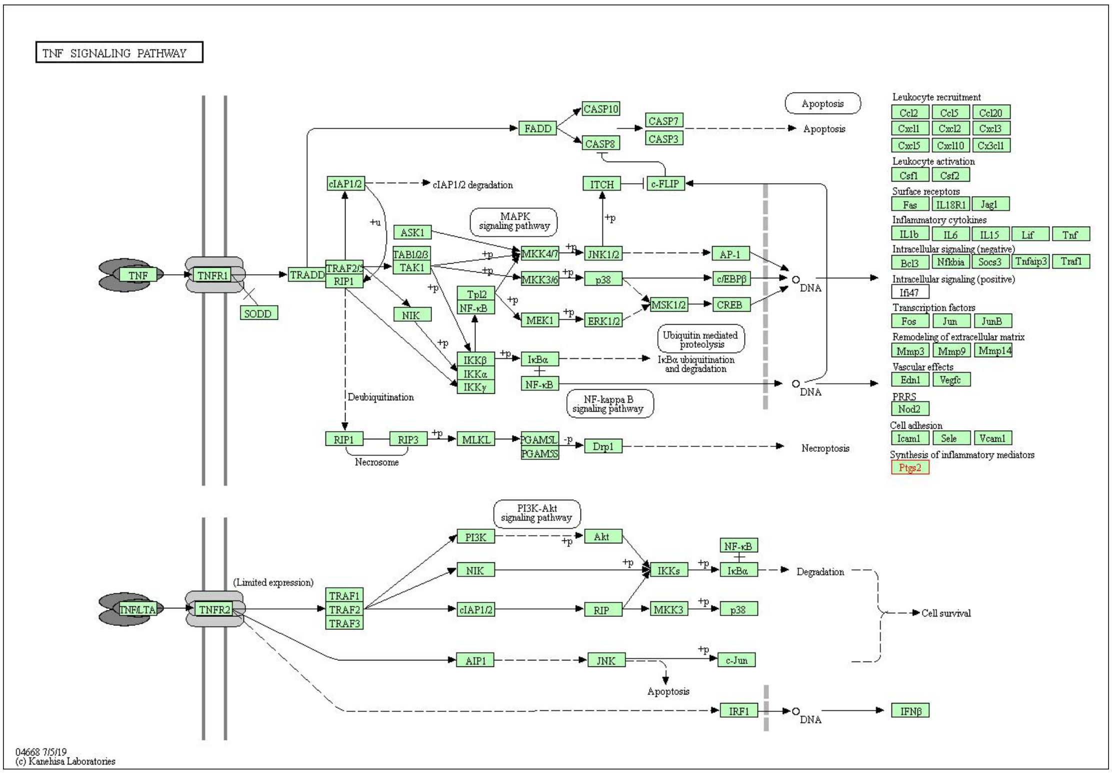

Supplementary Figure 2 TNF signaling pathway

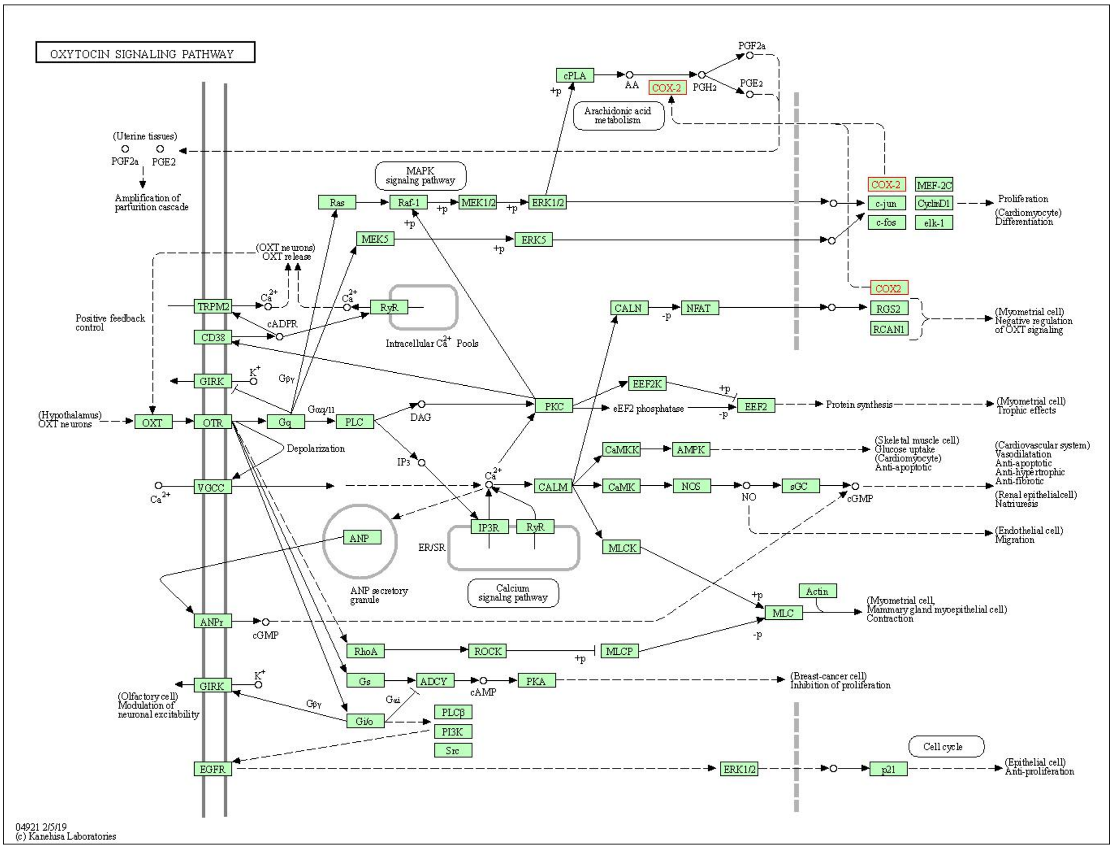

Supplementary Figure 3 Oxytocin signaling pathway

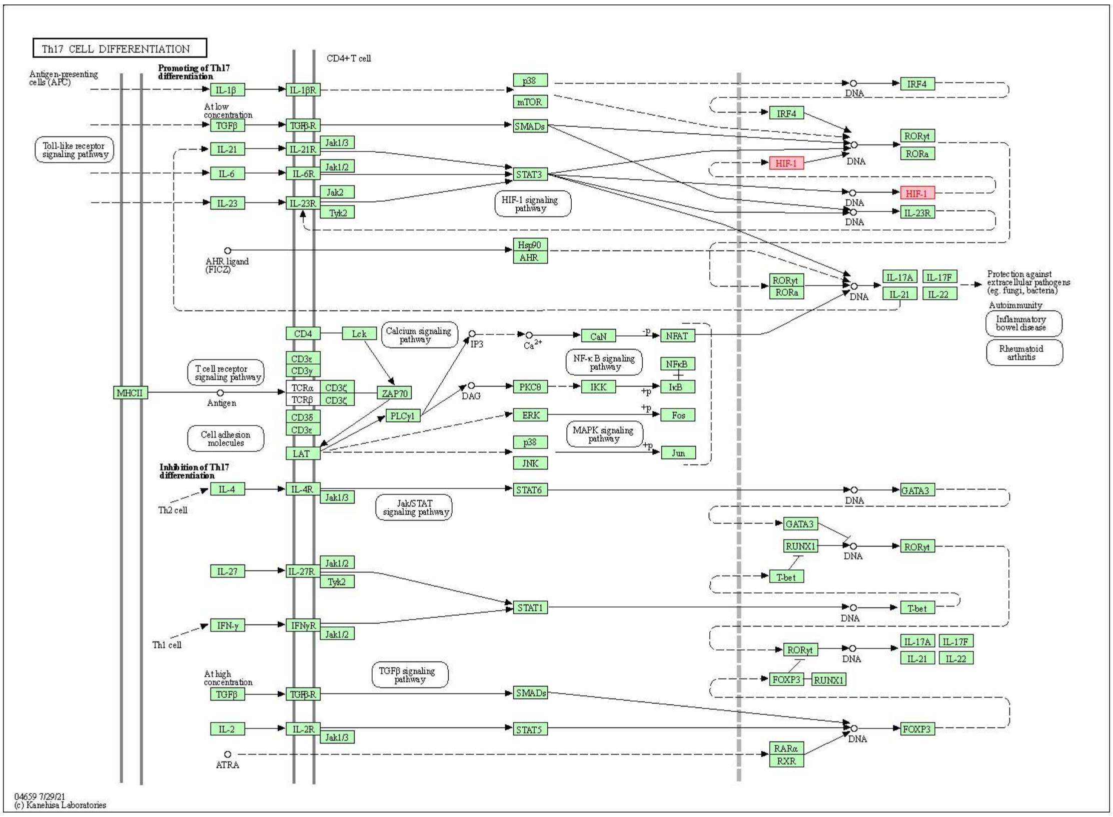

Supplementary Figure 4 Th17 cell differentiation

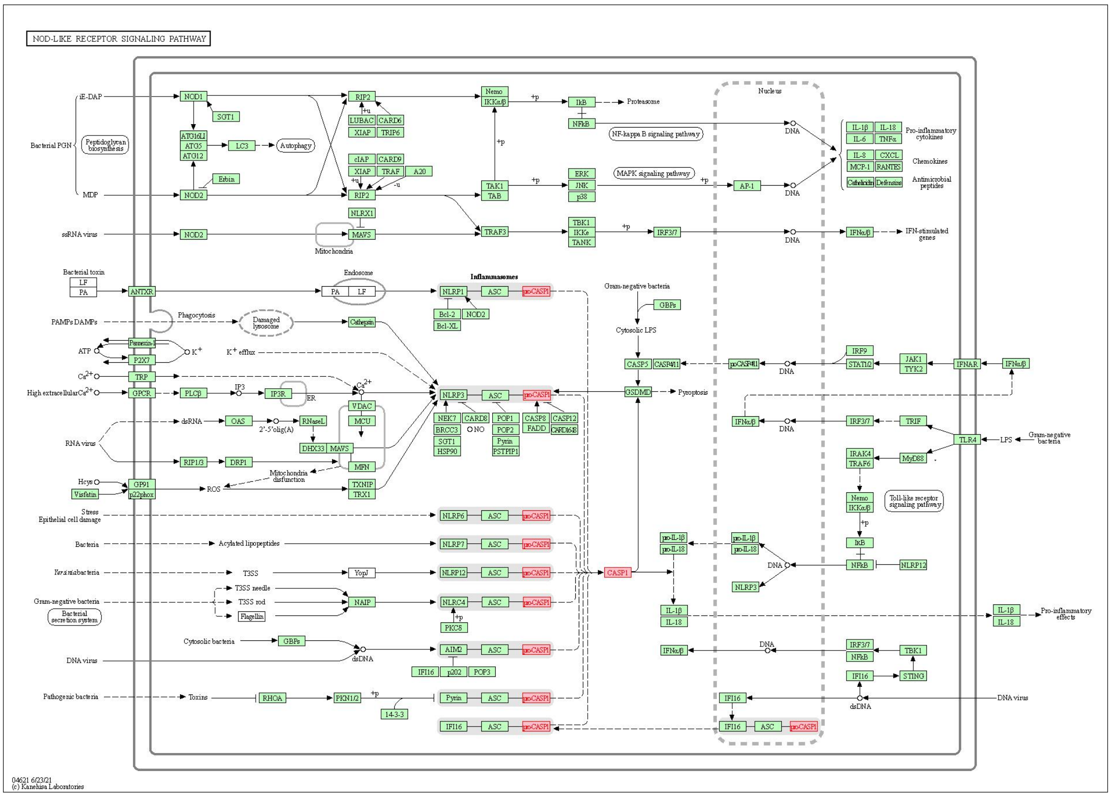

Supplementary Figure 5 NOD-like receptor signaling pathway

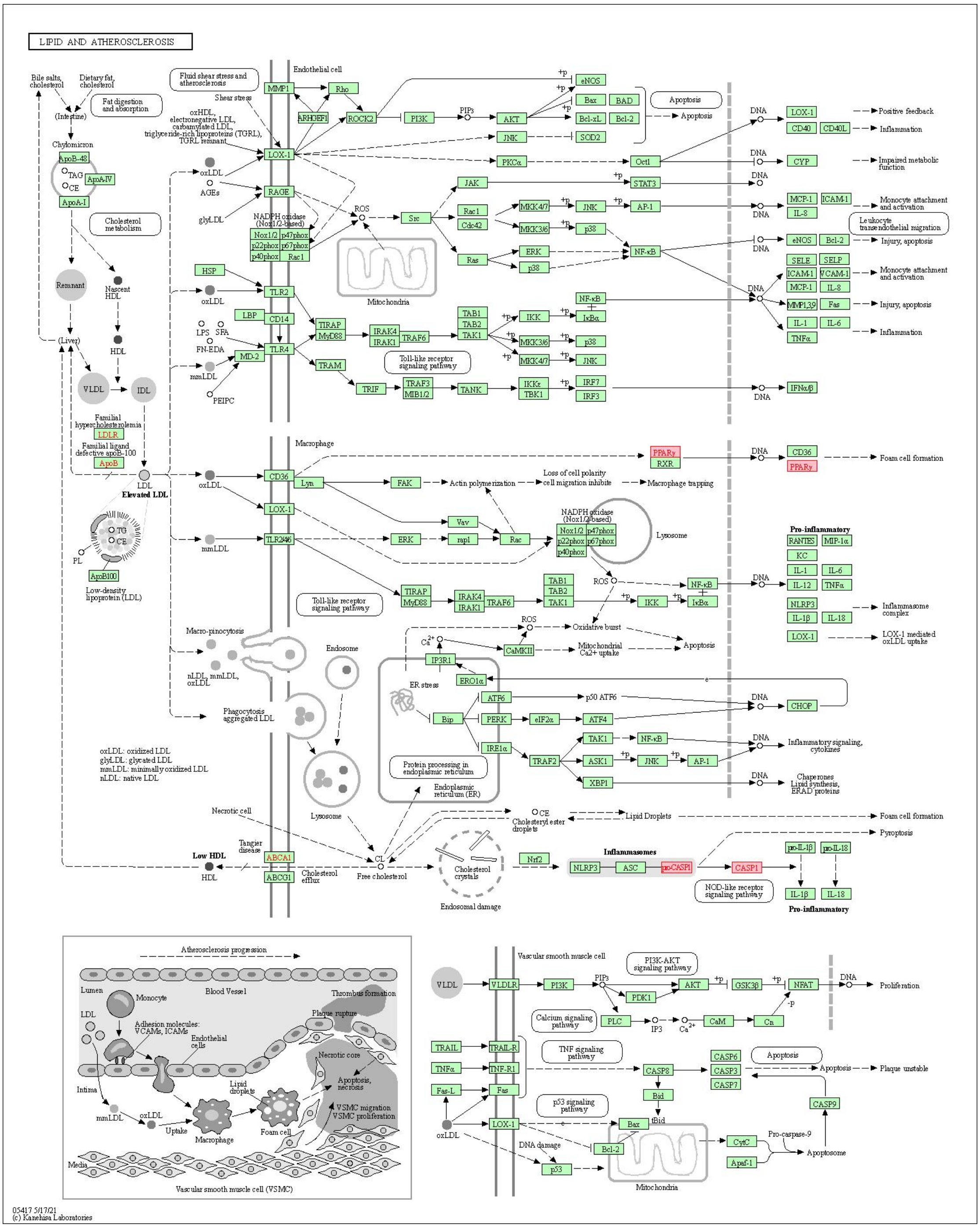

Supplementary Figure 6 Lipid and atherosclerosis

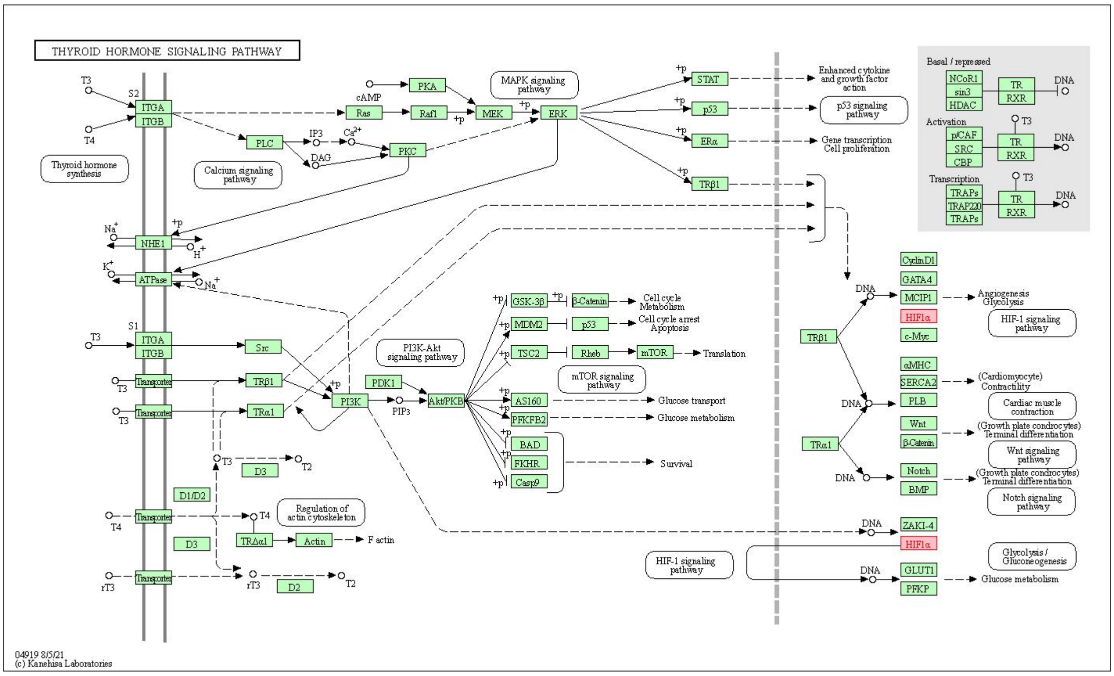

Supplementary Figure 7 Thyroid hormone signaling pathway
